# Detection of antibiotic resistance is essential for gonorrhoea point-of-care testing: a mathematical modelling study

**DOI:** 10.1186/s12916-017-0881-x

**Published:** 2017-07-26

**Authors:** Stephanie M. Fingerhuth, Nicola Low, Sebastian Bonhoeffer, Christian L. Althaus

**Affiliations:** 1 Institute of Integrative Biology, ETH Zurich, Zurich, 8092 Switzerland; 2 Institute of Social and Preventive Medicine, University of Bern, Bern, 3012 Switzerland

**Keywords:** Gonorrhoea, Bacterial drug resistance, Point-of-care testing, Mathematical model, Sexually transmitted infection, Epidemiology

## Abstract

**Background:**

Antibiotic resistance is threatening to make gonorrhoea untreatable. Point-of-care (POC) tests that detect resistance promise individually tailored treatment, but might lead to more treatment and higher levels of resistance. We investigate the impact of POC tests on antibiotic-resistant gonorrhoea.

**Methods:**

We used data about the prevalence and incidence of gonorrhoea in men who have sex with men (MSM) and heterosexual men and women (HMW) to calibrate a mathematical gonorrhoea transmission model. With this model, we simulated four clinical pathways for the diagnosis and treatment of gonorrhoea: POC test with (POC+R) and without (POC−R) resistance detection, culture and nucleic acid amplification tests (NAATs). We calculated the proportion of resistant infections and cases averted after 5 years, and compared how fast resistant infections spread in the populations.

**Results:**

The proportion of resistant infections after 30 years is lowest for POC+R (median MSM: 0.18*%*, HMW: 0.12*%*), and increases for culture (MSM: 1.19*%*, HMW: 0.13*%*), NAAT (MSM: 100%, HMW: 99.27*%*), and POC−R (MSM: 100%, HMW: 99.73*%*). Per 100 000 persons, NAAT leads to 36 366 (median MSM) and 1228 (median HMW) observed cases after 5 years. Compared with NAAT, POC+R averts more cases after 5 years (median MSM: 3353, HMW: 118). POC tests that detect resistance with intermediate sensitivity slow down resistance spread more than NAAT. POC tests with very high sensitivity for the detection of resistance are needed to slow down resistance spread more than by using culture.

**Conclusions:**

POC with high sensitivity to detect antibiotic resistance can keep gonorrhoea treatable longer than culture or NAAT. POC tests without reliable resistance detection should not be introduced because they can accelerate the spread of antibiotic-resistant gonorrhoea.

**Electronic supplementary material:**

The online version of this article (doi:10.1186/s12916-017-0881-x) contains supplementary material, which is available to authorized users.

## Background

Antibiotic resistance is a major challenge for the management of gonorrhoea globally: extended-spectrum cephalosporins are the last antibiotic class remaining for empirical treatment of gonorrhoea [1, 2], and 42 countries have already reported *Neisseria gonorrhoeae* strains with decreased susceptibility against them [2]. The first strain with high-level resistance to the recommended combination therapy with ceftriaxone and azithromycin was recently described [3]. With an estimated 78 million new gonorrhoea cases each year [4], new control strategies are urgently needed before gonorrhoea becomes untreatable.

Conventional diagnostic tests for gonorrhoea, such as nucleic acid amplification tests (NAATs) and culture, are not sufficient to control antibiotic resistance. Commercially available NAATs, the most commonly used diagnostic gonorrhoea tests in high-income countries, cannot detect antibiotic resistance [5, 6]. Cultures of *N. gonorrhoeae* can be used to determine antibiotic-resistance profiles, but reliable results depend on stringent collection and transport of specimens [7]. Culture and susceptibility testing need several days to deliver results [8]. NAATs can deliver results in a few hours [9], but in routine use specimens might be tested in batches [10] and NAAT results might be delivered only after several days [11]. While symptomatic gonorrhoea patients usually receive empirical treatment at their first visit, asymptomatic patients might have to return for treatment. Loss to follow-up and the further spread of resistant infections can result.

Point-of-care (POC) tests promise to help control antibiotic resistance [12]. POC tests provide results rapidly and allow informed clinical decisions about treatment at the first visit of a patient. POC tests, therefore, reduce the time to treatment and avoid loss to follow-up. A modelling study suggested that POC tests can reduce gonorrhoea prevalence if no antibiotic resistance is present in the population [13]. Though not yet commercially available [12], POC tests that detect resistance promise to reduce the use of antibiotics [14] and to spare last-line antibiotics through individually tailored treatment [15, 16]. One modelling study illustrated that individualised treatment could slow down the spread of resistance as much as combination therapy [17]. However, reduced time to treatment and increased follow-up with POC tests might increase the rate of gonorrhoea treatment. Since higher treatment rates can lead to the faster spread of resistance [18, 19], POC tests might increase resistance levels. We extended a previously developed mathematical model of gonorrhoea transmission [19] to compare the effects of current conventional tests (culture and NAATs) with POC tests that reduce time to treatment and loss to follow-up. We investigated the potential impact of POC tests on resistance and on the number of gonorrhoea cases for a population at high risk of infection [20], men who have sex with men (MSM), and a population at lower risk of infection, heterosexual men and women (HMW).

## Methods

**Fig. 1:**
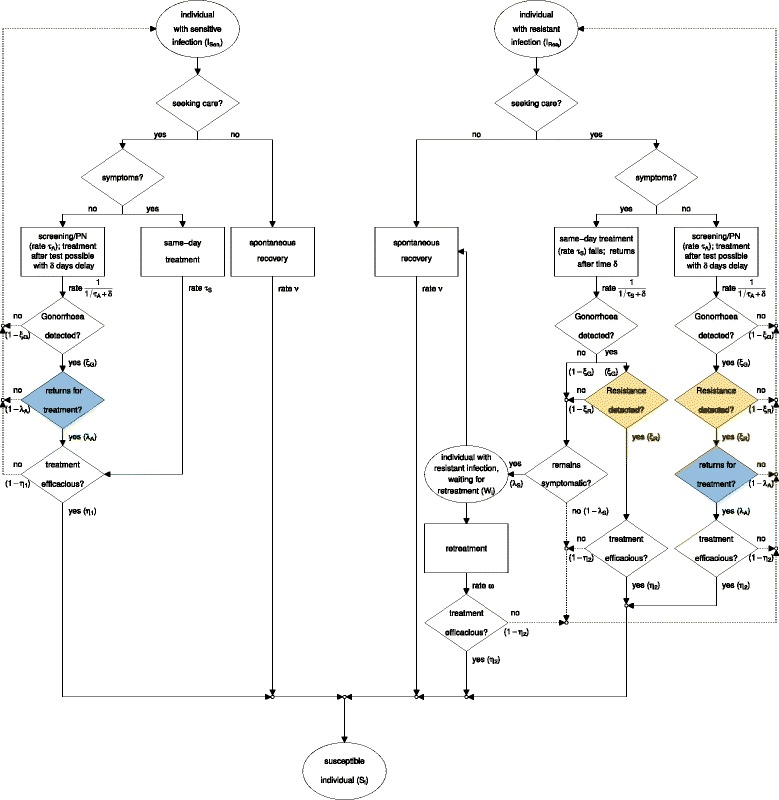
Clinical pathway for the testing and treatment of gonorrhoea infections. Gonorrhoea patients can recover spontaneously or seek care for their infection. Depending on the parameter values of *δ*, *λ*_*A*_, and *ξ*_*R*_ (Table 2), patients are tested with culture, NAAT, POC−R, or POC+R when they seek care. In the NAAT and POC−R scenarios, “Resistance detected?” (*yellow*) defaults to “no”. In POC−R and POC+R, “returns for treatment?” (*blue*) defaults to “yes”. In the culture scenario, the flowchart is followed as shown. *Dashed arrows* indicate that individuals remain infected. *NAAT* nucleic acid amplification test, *POC* point-of-care, *POC*+*R* POC test with resistance detection, *P* *O* *C*−*R* POC test without resistance detection, *PN* partner notification

We developed a mathematical model that describes transmission of antibiotic-sensitive and -resistant gonorrhoea, clinical pathways for diagnostic testing with culture, NAAT, or POC, and treatment with first- and second-line antibiotics (Additional file 1: Section Model). Here we describe the model, focusing on testing and treatment of gonorrhoea (Fig. 1 and Table 1).

**Table 1:** Gonorrhoea testing and treatment parameters and their default values

Parameter	Description (unit)	Baseline	Culture	NAAT	POC+R	POC−R
*τ*_*S*_	Rate at which symptomatic individuals seek care (y^−1^)	Variable ^a^	See baseline	See baseline	See baseline	See baseline
*τ*_*A*_	Rate at which asymptomatic individuals seek care (y^−1^)	Variable ^a^	See baseline	See baseline	See baseline	See baseline
*ξ*_*G*_	Test sensitivity to detect gonorrhoea	99% ^b^	See baseline	See baseline	See baseline	See baseline
*ξ*_*R*_	Test sensitivity to detect resistance against the first-line antibiotic	Any value	99% ^c^	0% ^d^	99% ^c^	0% ^d^
*η*_1_, *η*_2_	Efficacy of first-line (1) or second-line (2) antibiotic	99% ^e^	See baseline	See baseline	See baseline	See baseline
*δ*	Average time after test individuals return for treatment (days)	7 ^f^	See baseline	See baseline	0 ^d^	0 ^d^
1/*ω*	Average time individuals with resistant gonorrhoea wait for re-treatment (days)	7 ^c^	See baseline	See baseline	See baseline	See baseline
*λ*_*A*_	Fraction of asymptomatic individuals who return for treatment	90% ^c^	See baseline	See baseline	100% ^d^	100% ^d^
*λ*_*S*_	Fraction of symptomatic individuals who remain symptomatic after failed treatment	90% ^c^	See baseline	See baseline	See baseline	See baseline
*ψ*	Fraction of successfully treated individuals who were symptomatic at baseline	60% ^f^	–	–	–	–

Unless a value is set by definition, all values listed are default values and are varied in sensitivity analyses. Baseline: Resistance-free scenario (corresponds to scenario where culture or NAAT is used; *ξ*_*R*_ can take any value since there is no resistance to detect). Culture, NAAT, POC−R, or POC+R refer to scenarios after resistance is introduced

*NAAT* nucleic acid amplification test, *POC* point-of-care, *POC*+*R* POC test with resistance detection, *POC*−*R* POC test without resistance detection

^a^Derived

^b^[35]

^c^Assumption

^d^By definition

^e^[23, 36]

^f^[11]

### Basic model structure

The model is based on our previously published compartmental model of gonorrhoea transmission and resistance spread [19]. The model describes a population with two sexual activity classes *i*∈*C*, where *C*={*L, H*} indicates that there are two sexual activity classes *L* and *H* with low and high partner change rates. The model incorporates sexual mixing between the sexual activity classes, sexual behaviour change, migration in and out of the population, and gonorrhoea transmission. Individuals in the population can be susceptible to infection, *S*_*i*_, infected with antibiotic-sensitive gonorrhoea, $\phantom {\dot {i}\!}I_{\text {Sen}_{i}}$, infected with gonorrhoea resistant to the first-line antibiotic, $\phantom {\dot {i}\!}I_{\text {Res}_{i}}$, or infected with gonorrhoea resistant to the first-line antibiotic and waiting for re-treatment, *W*_*i*_. Depending on the parameters for sexual behaviour, transmission, and gonorrhoea natural history (Additional file 1: Table S2), the model describes a population of MSM or HMW.

### Gonorrhoea testing and treatment

#### Antibiotic-sensitive gonorrhoea

Individuals infected with antibiotic-sensitive gonorrhoea, $\phantom {\dot {i}\!}I_{\text {Sen}_{i}}$, can recover spontaneously at rate *ν* or seek care (Fig. 1, left). Symptomatic care-seekers receive treatment on the same day at rate *τ*_*S*_. Asymptomatic care-seekers, i.e., those who are screened for gonorrhoea or were notified through an infected partner, are tested at rate *τ*_*A*_. Gonorrhoea is detected with sensitivity *ξ*_*G*_. On average, a fraction *λ*_*A*_ of asymptomatic individuals returns for treatment after *δ* days. The treatment rate for asymptomatic individuals is approximated by 1/(1/*τ*_*A*_+*δ*), the inverse of the average time until individuals are tested, 1/*τ*_*A*_, and the time until they return for treatment, *δ*. Both symptomatic and asymptomatic individuals are treated with a first-line antibiotic that has treatment efficacy *η*_1_. We assumed that individuals whose treatment was inefficacious remain infected and do not seek care again immediately. This assumption reflects the notion that treatment failure of antibiotic-sensitive gonorrhoea is most likely to occur in pharyngeal infections, which are usually asymptomatic [21].

#### Antibiotic-resistant gonorrhoea

Individuals infected with gonorrhoea resistant to the first-line antibiotic, $\phantom {\dot {i}\!}I_{\text {Res}_{i}}$, can also recover spontaneously at rate *ν* (Fig. 1, right). Asymptomatic care-seekers that return for treatment (fraction *λ*_*A*_) receive treatment with the second-line antibiotic at rate 1/(1/*τ*_*A*_+*δ*) if both gonorrhoea (sensitivity *ξ*_*G*_) and resistance (sensitivity *ξ*_*R*_) are detected. Symptomatic care-seekers receive the first-line antibiotic as treatment on the same day, but remain infected due to resistance and return for treatment after *δ* days. At their second visit, symptomatic care-seekers receive the second-line antibiotic if both gonorrhoea (sensitivity *ξ*_*G*_) and resistance (sensitivity *ξ*_*R*_) are detected. If either test fails, they do not receive the second-line antibiotic. If they remain symptomatic (fraction *λ*_*S*_), they wait for re-treatment in compartment *W*_*i*_, where they either receive re-treatment with the second-line antibiotic at rate *ω* or recover spontaneously at rate *ν*. The assumption that re-treatment occurs with the second-line antibiotic follows recommendations from the World Health Organization (WHO) [20] and the Centers for Disease Control (CDC) [22] to obtain a specimen for culture-based antibiotic resistance testing at a patient’s second visit. The second-line antibiotic has efficacy *η*_2_; individuals whose treatment is inefficacious remain infected and can recover spontaneously or seek care at a later point. De novo resistance to the first-line antibiotic or resistance to the second-line antibiotic are not considered in the model.

### Testing scenarios

We simulated clinical pathways of gonorrhoea patients who are tested with culture, NAAT, or POC test at their first visit by adapting the parameters *δ*, *λ*_*A*_, and *ξ*_*R*_ (Table 2). For culture, test results are not available immediately (*δ*_culture_>0), resistance can be detected (*ξ*_*R*,culture_>0), and asymptomatic infected individuals might not return for treatment (*λ*_*A*,culture_<1). For NAAT, test results are not available immediately (*δ*_NAAT_>0), resistance cannot be detected (*ξ*_*R*,NAAT_=0), and asymptomatic infected individuals might not return for treatment (*λ*_*A*,NAAT_<1). For POC, test results are available immediately (*δ*_POC_=0), all individuals are followed up (*λ*_*A*,POC_=1), and thus all individuals are treated at the first visit. We explore the impact of a POC test with (*ξ*_*R*,POC_>0, POC+R) and without resistance detection (*ξ*_*R*,POC_=0, POC−R); we use the term “POC” alone when *ξ*_*R*,POC_ is variable.

**Table 2:** Culture, NAAT, and POC testing scenarios are determined by the values of *δ*, *λ*_*A*_, and *ξ*_*R*_

Scenario	*δ*	*λ*_*A*_	*ξ*_*R*_
Culture	> 0	< 1	> 0
NAAT	> 0	< 1	= 0
POC	= 0	= 1	≥0
POC+R	= 0	= 1	> 0
POC−R	= 0	= 1	= 0

Culture, NAAT, and POC testing scenarios can be simulated with the same mathematical model by adapting the average time after test that individuals return for treatment (*δ*), the fraction of asymptomatic individuals who return for treatment (*λ*_*A*_), and the test sensitivity to detect resistance against the first-line antibiotic (*ξ*_*R*_)

*NAAT* nucleic acid amplification test, *POC* point-of-care, *POC + R* POC test with resistance detection, *POC − R* POC test without resistance detection

### Impact measures

We evaluated the impact of a testing scenario by calculating the proportion of resistant infections among all infections, observed cases averted, and the rate at which resistance spreads, compared with another testing scenario. We measured the proportion of resistant infections up to 30 years after the introduction of resistance into the resistance-free baseline scenario. If applicable, we also calculated the time until resistance levels reached 5%, the level above which an antibiotic should not be used for empirical gonorrhoea treatment [23]. We defined observed cases averted as the difference between the cumulative incidence of observed cases (i.e., cases diagnosed and successfully treated at baseline; fraction *ϕ* [19]) using NAAT and the cumulative incidence of observed cases using culture or POC tests. We calculated the observed cases averted 5 years after the introduction of resistance. The rate at which resistance spreads describes how fast resistant infections replace sensitive infections in a human population [19]. We calculated the ratio of the rate of resistance spread between POC with different test sensitivities to detect resistance (*ξ*_*R*,POC_) and culture or NAAT scenarios (Additional file 1: Section Rate of resistance spread). If the ratio of the rate of resistance spread is >1, resistance spreads faster when using POC tests compared with other tests. If the ratio is <1, resistance spreads slower when using POC tests compared with other tests.

### Parameters

We used the parameters describing sexual behaviour, gonorrhoea transmission, natural history, and treatment from our previous model [19]. There, we estimated sexual behaviour parameters from the second British National Survey of Sexual Attitudes and Lifestyles (Natsal-2), which is a nationally representative population-based survey [24]. We calibrated all other parameters to yield prevalence and incidence rates within empirically observed ranges (Tables 3 and 4). We assumed that the empirically observed values refer to a period during which treatment was mostly effective and thus, used model simulations without resistance for the calibration. For this study, we used subsets of 1000 calibrated parameter sets from the previous study to simulate MSM and HMW populations (prevalence and incidence rates in Additional file 1: Figures S2 and S3). For each calibrated parameter set, we derived the care-seeking rate of asymptomatic (*τ*_*A*_) and symptomatic (*τ*_*S*_) individuals using the fraction of successfully treated individuals who were symptomatic at baseline *ϕ* (Additional file 1: Section Derivation of *τ*_A_ and *τ*_S_). We set default values for the testing and treatment parameters (*ψ*, *ξ*_*G*_, *ξ*_*R*_, *η*_1_, *η*_2_, *δ*, *ω*, *λ*_*A*_, and *λ*_*S*_) guided by the literature (Table 1).

**Table 3:** Gonorrhoea prevalence and incidence in baseline scenario (before resistance introduced) for men who have sex with men

	Range used for calibration	Baseline median (IQR)
Prevalence low activity class (%)	0–2.79	0.59 (0.42–0.79)
Prevalence high activity class (%)	1.19–100	27.64 (23.25–31.91)
Prevalence total population (%)	1.19–2.79	2.09 (1.69–2.43)
Incidence total population (100 000 persons^−1^ y^−1^)	5880–7190	6493.49 (6192.89–6842.70)

The prevalence and incidence ranges used for calibration for men who have sex with men were based on the Health in Men Study in Australia [37]. The baseline median and IQR are based on the simulation results of 1000 calibrated parameter sets. The upper and lower bounds of the calibration range for the low and high sexual activity classes were set to the lower and upper bounds for the total population. The calibration is detailed in [19]

*IQR* interquartile range

**Table 4:** Gonorrhoea prevalence and incidence in baseline scenario (before resistance introduced) for heterosexual men and women

	Range used for calibration	Baseline median (IQR)
Prevalence low activity class (%)	0–0.38	0.12 (0.09–0.15)
Prevalence high activity class (%)	0.16–100	2.14 (1.71–2.60)
Prevalence total population (%)	0.16–0.38	0.25 (0.21–0.3)
Incidence total population (100 000 persons^−1^ y^−1^)	120–360	222.13 (172.19–283.54)

The prevalence and incidence ranges used for calibration for heterosexual men and women were based on the National Health and Nutrition Examination Survey [38] and surveillance data [39], both from CDC. The baseline median and IQR are based on the simulation results of 1000 calibrated parameter sets. The upper and lower bounds of the calibration range for the low and high sexual activity classes were set to the lower and upper bounds for the total population. The calibration is detailed in [19]

*CDC* Centers for Disease Control, *IQR* interquartile range

### Sensitivity Analyses

We performed sensitivity analyses to confirm that our model results are robust in scenarios with different properties of tests (*ξ*_*G*_ and *ξ*_*R*_), antibiotics (*η*_1_ and *η*_2_), and populations and clinics (*δ*, *ω*, *λ*_*A*_, and *λ*_*S*_). First, we performed sensitivity analyses of the observed cases averted with regard to changes in both the fraction of asymptomatic individuals who return for treatment at baseline (*λ*_*A*,baseline_) and the fraction of successfully treated individuals who were symptomatic at baseline (*ψ*), as well as to changes in single testing and treatment parameters (*ξ*_*G*_, *ξ*_*R*_, *λ*_*A*,baseline_, *λ*_*S*_, *ψ*, *δ*_baseline_, and *ω*). Second, we evaluated the sensitivity of the ratio of resistance spread with regard to changes in the test sensitivity to detect resistance against the first-line antibiotic when using POC (*ξ*_*R*,POC_), the fraction of asymptomatic individuals who return for treatment at baseline (*λ*_*A*,baseline_), and the fraction of successfully treated individuals who were symptomatic at baseline (*ψ*). Third, we tested the sensitivity of our model results to the assumption that the test sensitivity to detect *N. gonorrhoeae* is 99% for culture testing. For this, we simulated an alternative scenario where culture has a lower test sensitivity to detect *N. gonorrhoeae* and only culture is used at baseline (*ξ*_*G*,baseline_=*ξ*_*G*,culture_=90%, all other parameters as in Table 1).

### Simulation

For each parameter set, we first simulated a resistance-free baseline scenario where either culture or NAAT is used (*δ*>0, *λ*_*A*_<1). We simulated the baseline scenario until it reached equilibrium using the function runsteady in the package rootSolve [25] from the R language and software environment for statistical computing [26]. Next, we introduced resistant strains by converting 0.1*%* of all sensitive infections into resistant infections. We then set the parameter *ξ*_*R*_ to reflect the different testing scenarios (culture, NAAT, POC+R, or POC−R). For POC tests, we additionally set *δ*=0 and *λ*_*A*_=1. Finally, we simulated the model using the function lsoda from the R package deSolve [27].

## Results

### Proportion of resistant infections

**Fig. 2:**
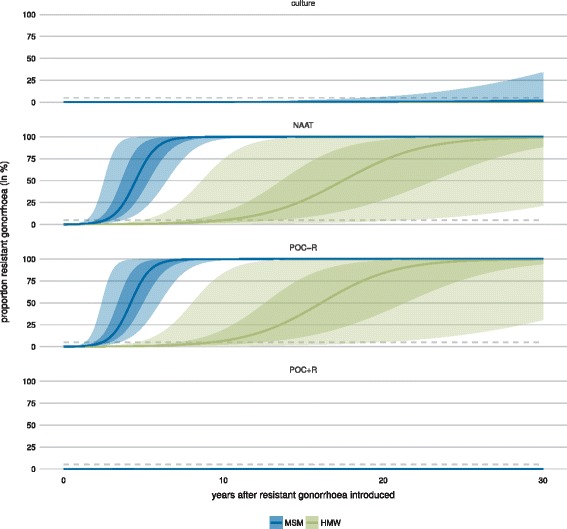
Time line of the proportion of resistant gonorrhoea infections when using culture, NAAT, POC−R, or POC+R. The proportion of resistant infections remains lowest when POC+R is used, followed by culture. The proportion of resistant infections exceeds the 5% threshold (*dashed lines*) marginally earlier with POC−R than with NAAT. The *continuous lines* give the median proportion of resistant infections over all simulations. *Shaded areas* indicate that 50% or 95% of all simulations lie within this range. *HMW* heterosexual men and women, *MSM* men who have sex with men, *NAAT* nucleic acid amplification test, *POC* point-of-care, *POC + R* POC test with resistance detection, *POC − R* POC test without resistance detection

We determined the proportion of gonorrhoea infections resistant to the first-line antibiotic for up to 30 years after the introduction of resistance (Fig. 2). The proportion of resistant infections remains lowest when POC+R is used (MSM: median 0.18*%* after 30 years, interquartile range (IQR) 0.17–0.21%; HMW: 0.12*%*, 0.11–0.12%). The proportion of resistant infections also remains low with culture (MSM: 1.19*%*, 0.68–3.59%, HMW: 0.13*%*, 0.12–0.15%). In contrast, resistant infections largely replace sensitive infections after 30 years using NAAT (MSM: 100%, 100–100%, HMW: 99.27*%*, 88.54–99.97%) and POC−R (MSM: 100%, 100–100%, HMW: 99.73*%*, 94.30–99.99%). The proportion of resistant infections exceeds the 5% resistance threshold (Fig. 2, dashed line) marginally earlier when POC−R is used (MSM: median <2.42, IQR 2.00–2.92 years, HMW: <9.25, 7.25–12.25 years) than when NAAT is used (MSM: <2.58, 2.08–3.08 years, HMW: <10.08, 7.83−13.33 years). Overall, POC+R performs best in keeping the proportion of resistant infections low and POC−R performs worst.

We tested the sensitivity of the proportion of resistant infections to the assumption that the test sensitivity to detect *N. gonorrhoeae* is 99% for culture testing (*ξ*_*G*,culture_=99*%*) by simulating an alternative scenario where culture has a lower test sensitivity to detect *N. gonorrhoeae* and only culture is used at baseline (with *ξ*_*G*,baseline_=*ξ*_*G*,culture_=90*%* and all other values as in Table 1; Additional file 1: Figure S12). The proportions of resistant infections after 30 years are higher when *ξ*_*G*,baseline_=*ξ*_*G*,culture_=90*%* for the culture scenario (MSM: median 3.18*%*, IQR 1.51–11.33%; HMW: 0.16*%*, 0.14–0.20%) and slightly higher for the other tests (NAAT MSM: 100%, 100–100%, HMW: 99.71%, 94.04–99.99%; POC−R MSM: 100%, 100–100%, HMW: 99.91%, 97.38–100.00%; POC+R MSM: 0.19*%*, 0.17–0.22%, HMW: 0.12*%*, 0.11–0.12%). The proportion of resistant infections exceeds 5% slightly earlier when *ξ*_*G*,baseline_=*ξ*_*G*,culture_=90*%* in the POC−R scenario (MSM: median <2.25 years, IQR 1.83–2.67 years, HMW: <8.58 years, 6.75–11.33 years) and NAAT scenario (MSM: <2.42 years, 2.00–2.83 years, HMW: 9.33 years, 7.25–12.33 years).

### Observed cases averted

**Fig. 3:**
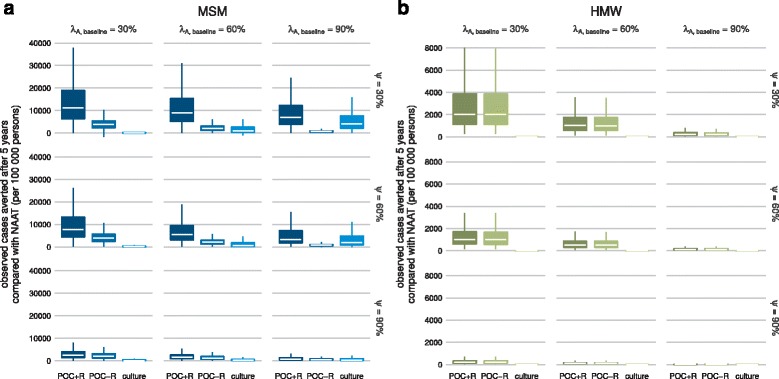
Two-dimensional sensitivity analysis of observed cases averted (per 100 000 persons) after 5 years. The sensitivity analysis is performed with respect to the fraction of asymptomatic individuals who return for treatment at baseline (*λ*_*A*,baseline_) and the fraction of successfully treated individuals who were symptomatic at baseline (*ψ*), for **a** MSM and **b** HMW. The central right plot of each panel shows the default scenario (*λ*_*A*,baseline_=90*%* and *ψ*=60*%*). *Lower* and *upper* bounds in a box indicate the first and third quartiles. The *bar* in a box indicates the median and the *whiskers* span 1.5 times the interquartile range. Outliers are not shown for clarity. *HMW* heterosexual men and women, *MSM* men who have sex with men, *NAAT* nucleic acid amplification test, *POC* point-of-care, *POC + R* POC test with resistance detection, *POC − R* POC test without resistance detection

We calculated the observed cases averted (per 100 000 persons) after 5 years using culture, POC+R, or POC−R in comparison with NAAT (Fig. 3). For the default values (*λ*_*A*,baseline_=90*%* and *ψ*=60*%*), using NAAT leads to a median of 36 366 (IQR 33 789–39 692) observed cases after 5 years for MSM and 1228 (927–1610) for HMW. Culture averts 1876 (740–4919) cases in MSM and 3 (1–7) in HMW compared with NAAT. POC+R averts even more cases than culture in both MSM (3353, 1697–7259) and HMW (118, 69–198). POC−R averts fewer cases than culture in MSM (772, 452–1119), but about the same as POC+R in HMW (115, 68–190).

First, we performed sensitivity analyses of the observed cases averted with regard to changes in both the fraction of asymptomatic individuals who return for treatment at baseline (*λ*_*A*,baseline_) and the fraction of successfully treated individuals who were symptomatic at baseline (*ψ*) (Fig. 3). For culture, increasing the fraction of asymptomatic individuals who return for treatment at baseline (*λ*_*A*,baseline_) and decreasing the fraction of successfully treated individuals who were symptomatic at baseline (*ψ*) increases the median observed cases averted. For POC+R, decreasing *λ*_*A*,baseline_ and decreasing *ψ* leads to an increase in the median observed cases averted. For POC−R, a decreasing value of *λ*_*A*,baseline_ and an intermediate value of *ψ* results in an increase in median averted cases. For all combinations of *λ*_*A*,baseline_ and *ψ* in both MSM and HMW, POC+R averts more cases at the median than culture.

Second, we performed sensitivity analyses of the observed cases averted with regard to changes in single testing and treatment parameters. Decreasing the test sensitivity to detect resistance against the first-line antibiotic (*ξ*_*R*,POC_ or *ξ*_*R*,culture_, Additional file 1: Figure S6), the average time after test individuals return for treatment (*δ*_baseline_, Additional file 1: Figure S10) or the average time individuals with resistant gonorrhoea wait for re-treatment (1/*ω*, Additional file 1: Figure S11) leads to a decrease in the median observed cases averted in POC+R and culture for both MSM and HMW. Decreasing the fraction of symptomatic individuals who remain symptomatic after failed treatment (*λ*_*S*_, Additional file 1: Figure S8) or the fraction of successfully treated individuals who were symptomatic at baseline (*ψ*, Additional file 1: Figure S9) leads to an increase in the median observed cases averted when using POC+R and culture for both MSM and HMW. Decreasing the test sensitivity to detect gonorrhoea (*ξ*_*G*_, Additional file 1: Figure S5) or the fraction of asymptomatic individuals who return for treatment at baseline (*λ*_*A*,baseline_, Additional file 1: Figure S7) leads to an increase in the median observed cases averted when using POC+R, but to a decrease in the median observed cases averted when using culture. For the same parameter values, POC+R averts more cases at the median than culture in both MSM and HMW.

Third, we simulated an alternative scenario where culture has a lower test sensitivity to detect *N. gonorrhoeae* and only culture is used at baseline (*ξ*_*G*,baseline_=*ξ*_*G*,culture_=90*%* and all other values as in Table 1; Additional file 1: Figure S13). In the alternative scenario, the number of observed cases averted after 5 years using POC+R compared with NAAT is larger than in the default scenario for MSM (median 4236, IQR 2161–8839 per 100 000 persons). The observed cases averted after 5 years compared with NAAT are similar to the default scenario when using POC+R for HMW (119, 72–195), POC−R for MSM (800, 452–1173) and HMW (116, 70–188), and culture for MSM (1863, 733–4908) and HMW (3, 1–7).

### Ratio of resistance spread

**Fig. 4:**
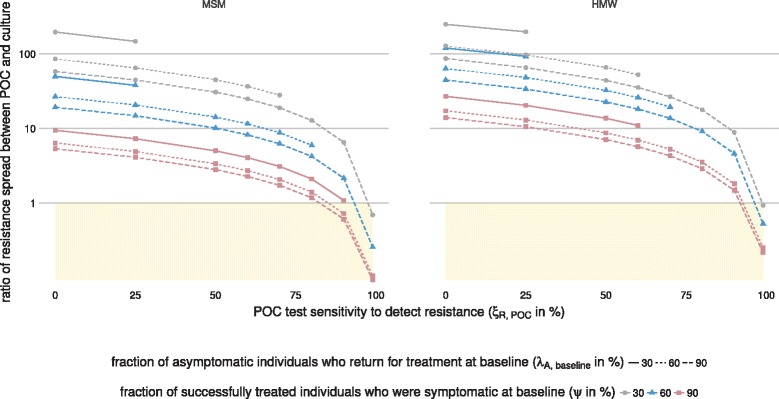
Three-dimensional sensitivity analysis of the ratio of resistance spread between POC and culture. Shown are the ratios of resistance spread for MSM and HMW for *ξ*_*R*,culture_=99*%* and different values of *ξ*_*R*,POC_, *λ*_*A*,baseline_, and *ψ* (POC−R: *ξ*_*R*,POC_=0, POC+R: *ξ*_*R*,POC_>0). The *shaded areas* indicate that resistance spread is slower when using POC than when using culture. For the default values (*ξ*_*R*,POC_=99*%*, *λ*_*A*,baseline_=90*%*, and *ψ*=60*%*), resistance spread is slower when using POC than when using culture. For most other values shown, using POC accelerates resistance spread. Each data point gives the median value over 1000 simulations (one per calibrated parameter set). Some calibrated parameter sets lead to the extinction of gonorrhoea in the simulation (Additional file 1: Figure S4). In these simulations, resistance did not spread and the ratio of resistance spread could not be calculated. Data points for these simulations were excluded from this figure since they would show the median ratio of resistance spread over less than 1000 simulations. Note that the *y*-axis is shown in logarithmic scale. *HMW* heterosexual men and women, *MSM* men who have sex with men, *POC* point-of-care, *POC + R* POC test with resistance detection, *POC − R* POC test without resistance detection

**Fig. 5:**
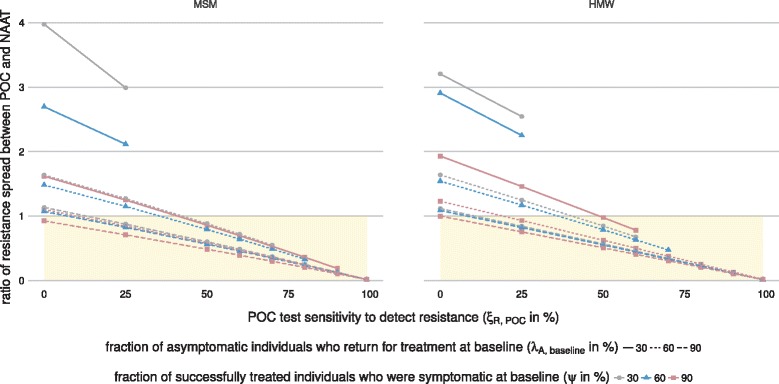
Three-dimensional sensitivity analysis of the ratio of resistance spread between POC and NAAT. Shown are the ratios of resistance spread for MSM and HMW for *ξ*_*R*,NAAT_=0*%* and different values of *ξ*_*R*,POC_, *λ*_*A*,baseline_, and *ψ* (POC−R: *ξ*_*R*,POC_=0, POC+R: *ξ*_*R*,POC_>0). The *shaded areas* indicate that resistance spread is slower when using POC than when using NAAT. For the default values (*ξ*_*R*,POC_=99*%*, *λ*_*A*,baseline_=90*%*, and *ψ*=60*%*) and most other values shown, resistance spread is slower when using POC than when using NAAT. Each data point gives the median value over 1000 simulations (one per calibrated parameter set). Some calibrated parameter sets lead to the extinction of gonorrhoea in the simulation (Additional file 1: Figure S4). In these simulations, resistance did not spread and the ratio of resistance spread could not be calculated. Data points for these simulations were excluded from this figure since they would show the median ratio of resistance spread over less than 1000 simulations. *HMW* heterosexual men and women, *MSM* men who have sex with men, *NAAT* nucleic acid amplification test, *POC* point-of-care, *POC + R* POC test with resistance detection, *POC − R* POC test without resistance detection

We determined the ratio of the rate of resistance spread between POC and culture (Fig. 4) and POC and NAAT (Fig. 5). For the default values (*ξ*_*R*,culture_=99*%*, *ξ*_*R*,NAAT_=0*%*, *ξ*_*R*,POC_=99*%*, *λ*_*A*,baseline_=90*%*, and *ψ*=60*%*), resistance spreads more slowly with POC compared with culture or NAAT.

First, we tested the sensitivity of the ratio of resistance spread to changes in the test sensitivity of POC to detect resistance (*ξ*_*R*,POC_). Decreasing *ξ*_*R*,POC_ can result in a faster spread of resistance with POC. A slight decrease in *ξ*_*R*,POC_ to 80–95% leads to faster resistance spread with POC compared with culture (Fig. 4). In contrast, only very low values of *ξ*_*R*,POC_ result in a faster resistance spread for POC compared with NAAT (Fig. 5).

Second, we tested the sensitivity of the ratio of resistance spread to changes in the fraction of asymptomatic individuals who return for treatment at baseline (*λ*_*A*,baseline_) and the fraction of successfully treated individuals who were symptomatic at baseline (*ψ*). Increasing *λ*_*A*,baseline_ leads to a lower ratio of resistance spread between POC and culture (Fig. 4) and POC and NAAT (Fig. 5). Similarly, increasing *ψ* leads to a lower ratio of resistance spread between POC and culture (Fig. 4) and POC and NAAT (Fig. 5).

Third, we simulated an alternative scenario where culture has a lower test sensitivity to detect *N. gonorrhoeae* and only culture is used at baseline (*ξ*_*G*,baseline_=*ξ*_*G*,culture_=90*%* and all other values as in Table 1; Additional file 1: Figure S14). If the ratio of resistance spread between POC and culture is above 1 in the default scenario, the ratio of resistance spread between POC and culture is smaller with the alternative scenario than with the default scenario. In contrast, if the ratio of resistance spread between POC and culture is below 1 with the default scenario, the ratio of resistance spread between POC and culture is larger with the alternative scenario than with the default scenario. The ratio of resistance spread between POC and NAAT is less affected by a different value of *ξ*_*G*,baseline_.

## Discussion

Using a mathematical transmission model, we compared the expected impact of POC tests on gonorrhoea cases and antibiotic resistance with conventional tests, culture and NAAT. We found that POC tests that detect antibiotic resistance with a sensitivity of 99% avert more gonorrhoea cases than any other test across all simulated settings. Additionally, we found that POC tests can slow down the spread of resistance if their test sensitivity to detect resistance is sufficiently high. If the test sensitivity of a POC test to detect resistance is higher than 0–40%, resistance spreads more slowly than with NAAT, and if POC sensitivity to detect resistance is higher than 80–95%, resistance spreads more slowly than with culture.

We captured the basic principles of the gonorrhoea testing and treatment process for culture, NAAT, and POC in a single model structure. The parameters describing the sexual behaviour and the natural history of gonorrhoea were estimated and calibrated in a previous study [19]. The default parameters that describe testing and treatment of gonorrhoea were based on literature values and are measurable. The model results are robust in sensitivity analyses (Figs. 3, 4 and 5, Additional file 1: Figures S5–S14). The model can be used to help design trials comparing different test strategies and guide the introduction of POC tests in the future.

Mathematical models generally depend on assumptions that should be taken into consideration when interpreting model results. We managed the complexity of our model with the following assumptions.

First, we did not consider test specificity. A low test specificity to detect resistance against the first-line antibiotic would result in increased use of the second-line antibiotic, and thus, simultaneously decrease the level of resistance against the first-line antibiotic and increase the level of resistance against the second-line antibiotic. Since we focused on resistance against the first-line antibiotic, we could not capture the impact of test specificity appropriately.

Second, our model does not include a change in antibiotic recommendations: undetected resistant infections are always treated with the first-line antibiotic, even if all infections in the population are resistant. This clinical pathway increases the average duration of resistant infections and possibly the observed cases. Whilst this is unlikely in high-income countries with good antibiotic resistance surveillance, it is not an unrealistic scenario in resource-poor settings without surveillance where 71–100% of gonococcal strains are resistant to fluoroquinolones [28]. In our model, MSM have a substantial level of resistant gonorrhoea infections after 5 years using NAAT. We expect that our model overestimates the observed cases using NAAT and the observed cases averted using culture and POC+R compared with a model including a change in antibiotic recommendations.

Third, we considered treatment with a single antibiotic although current treatment guidelines recommend a combination therapy with two antibiotics simultaneously [1, 7]. The model results are fully applicable to treatment with combination therapy if antibiotic-resistant gonorrhoea is interpreted as resistance against both antibiotics used for combination therapy.

Fourth, we investigated the effects of one test at a time and did not consider the effects of mixed testing. Our results, therefore, show only what the ideal effects of each test could be.

Fifth, we simplified the testing and treatment process. To compare the testing scenarios better, we did not model care-seeking and returning for treatment as separate processes, but approximated the overall treatment rates. In accordance with WHO [20] and CDC recommendations [22], we assumed that re-treatment of resistant infections occurs with the second-line antibiotic because a resistance profile has been determined after the second visit.

Sixth, for better comparability we assumed that culture, NAAT, and POC tests have the same sensitivity to detect gonorrhoea, even though culture has a lower sensitivity to detect rectal or pharyngeal gonorrhoea than molecular tests [29]. Our sensitivity analysis showed that a lower culture test sensitivity to detect gonorrhoea of 90% had a small effect on the model results.

Finally, it should be mentioned that the minimal POC test sensitivity to detect resistance that is necessary to slow down resistance spread will depend on the tests currently used, the setting, and population, and should be subject to validation.

Currently, there are no commercial POC tests that can detect antibiotic-resistant *N. gonorrhoeae* [12] and there remain challenges for their development. First, molecular POC test that detect resistance need molecular markers that reliably predict phenotypic resistance. So far only markers that predict resistance against some antibiotics are known [12, 30, 31]. Second, diagnostic tests need to deliver results fast to be considered POC. The fastest molecular diagnostic test for gonorrhoea that is commercially available takes 90 minutes [9, 32], which might be too long to wait for some patients. Finally, costs and training requirements for molecular tests have hindered their availability in low-income countries so far [33].

This study addresses two key questions for gonorrhoea control and resistance [34]. First, we investigated the potential impact of a POC test that detects antibiotic resistance (POC+R). We found that POC+R can slow resistance spread and reduce the number of gonorrhoea cases compared with culture or NAAT. The impact of POC+R is particularly strong when the fraction of asymptomatic individuals who return for treatment (*λ*_*A*,baseline_) and the fraction of successfully treated individuals who were symptomatic (*ψ*) were low before POC+R is introduced. However, when the POC test cannot detect resistance (POC−R), the benefits of POC are outweighed by accelerated resistance evolution. Because fewer patients are lost to follow-up, more patients are treated and more antibiotic treatment selects more strongly for antibiotic resistance. Since resistance cannot be detected, resistance levels increase and fewer cases are averted.

Second, we investigated the impact of POC tests in two populations with different levels of risk of gonorrhoea, MSM and HMW. We found that in both populations, POC tests with reliable resistance detection (POC+R) slow down the spread of resistance and avert the highest number of cases. POC tests without resistance detection (POC−R) avert about as many cases as POC+R in HMW, but clearly fewer cases than POC+R in MSM. Since resistance usually spreads faster in MSM [19], the faster spread of resistance caused by POC−R impacts the cases averted after 5 years in MSM, but not in HMW. POC tests that detect resistance reliably are crucial for both populations and both populations need culture-based surveillance of resistance to keep molecular markers for POC resistance detection updated.

## Conclusions

This modelling study addresses clinically relevant situations to evaluate the potential impact of gonorrhoea POC tests on antibiotic-resistant gonorrhoea and can guide the introduction of POC tests. POC tests with high sensitivity to detect resistance may replace culture-based diagnosis in clinical settings, as long as culture-based surveillance of antibiotic resistance is maintained to monitor resistance levels and to determine molecular markers for POC tests. POC tests with lower sensitivities to detect resistance should not replace culture-based diagnosis, but might have some advantages over NAAT. POC tests with low or no sensitivity to detect resistance should not be introduced, because POC tests without reliable resistance detection can accelerate the spread of antibiotic-resistant gonorrhoea.

## Additional file

Additional file 1 Full model description, model equations, figure depicting model, figure showing fraction of simulations in which gonorrhoea was eradicated, figures showing sensitivity analyses. (PDF 488 kb)

## Abbreviations

CDC: Centers for Disease Control
HMW: Heterosexual men and women
IQR: Interquartile range
MSM: Men who have sex with men
NAAT: Nucleic acid amplification test
POC: Point-of-care
POC+R: POC test with resistance detection
POC−R: POC test without resistance detection
WHO: World Health Organization
