# Supplementary material for: Detection of antibiotic resistance is essential for gonorrhoea point-of-care testing: a mathematical modelling study
Source: BMC Med. 2017 Jul 26;15:142. doi: 10.1186/s12916-017-0881-x (PMC5530576; doi:10.1186/s12916-017-0881-x)
Supplement: Additional file 1 — Full model description, model equations, figure depicting model, figure showing fraction of simulations in which gonorrhoea was eradicated, figures showing sensitivity analyses. (PDF 488 kb) [file 12916_2017_881_MOESM1_ESM.pdf]

Antibiotic resistance detection is essential for gonorrhoea point-of-care testing: A mathematical modelling study.

Additional file 1

Stephanie M. Fingerhuth[1,2]*, Nicola Low[2], Sebastian Bonhoeffer[1], Christian L. Althaus[2]

[1] *Institute of Integrative Biology, ETH Zurich, Universitätsstrasse 16, 8092 Zurich, Switzerland*
[2] *Institute of Social and Preventive Medicine, University of Bern, Finkenhubelweg 11, 3012 Bern, Switzerland*
***Corresponding author**: stephanie.fingerhuth@env.ethz.ch

Contents

1 **Model** 2
2 **Derivation of τ_A and τ_S** 5
3 **Prevalence and incidence rates before resistance** 5
4 **Rate of resistance spread and ratio of resistance spread** 7
5 **Sensitivity analyses** 8
References 19

List of Tables

S1 Description of model variables. 4
S2 Description of model parameters. 4

List of Figures

S1 Structure of gonorrhoea transmission, testing and treatment model. 3
S2 Prevalence and incidence of diagnosed and treated infections in MSM before resistance is introduced 6
S3 Prevalence and incidence of diagnosed and treated infections in HMW before resistance is introduced 6
S4 Eradication of gonorrhoea 7
S5 Sensitivity analysis of observed cases averted regarding ξ_G 9
S6 Sensitivity analysis of observed cases averted regarding ξ_R 10
S7 Sensitivity analysis of observed cases averted regarding $\lambda_{A,\text{ baseline}}$ 11
S8 Sensitivity analysis of observed cases averted regarding λ_S 12
S9 Sensitivity analysis of observed cases averted regarding ψ 13
S10 Sensitivity analysis of observed cases averted regarding δ_{baseline} 14
S11 Sensitivity analysis of observed cases averted regarding $1/\omega$ 15
S12 Alternative scenario: timeline 16
S13 Alternative scenario: observed cases averted 17
S14 Alternative scenario: ratio of resistance spread 18

1. Model

We extended a gonorrhoea transmission model that describes the transmission and treatment of antibiotic-sensitive and -resistant gonorrhoea [1] to include testing for gonorrhoea and resistance (Fig. S1; Table S1, S2):

$$\begin{aligned}
\dot{S_i} =& -S_i\pi_i \sum_{j\in C} \rho_{ij}\beta_{ij}\frac{I_{Sen_j}+I_{Res_j}+W_j}{N_j} + \nu(W_i+I_{Sen_i}+I_{Res_i}) + \omega\eta_2 W_i + (\frac{1}{\frac{1}{\tau_A}+\delta}\xi_G\lambda_A\eta_1 + \tau_S\eta_1)I_{Sen_i} \\
&+ \frac{1}{\frac{1}{\tau_A}+\delta}\xi_G\xi_R\lambda_A\eta_2 I_{Res_i} + \frac{1}{\frac{1}{\tau_S}+\delta}\xi_G\xi_R\eta_2 I_{Res_i} - \alpha S_i + \alpha N_i - \gamma S_i + \gamma N_i \sum_{j\in C} S_j, \\
\dot{I}_{Sen_i} =& S_i\pi_i \sum_{j\in C} \rho_{ij}\beta_{ij}\frac{I_{Sen_j}}{N_j} - \nu I_{Sen_i} - \frac{1}{\frac{1}{\tau_A}+\delta}\xi_G\lambda_A\eta_1 I_{Sen_i} - \tau_S\eta_1 I_{Sen_i} - \alpha I_{Sen_i} - \gamma I_{Sen_i} + \gamma N_i \sum_{j\in C} I_{Sen_j}, \\
\dot{I}_{Res_i} =& S_i\pi_i \sum_{j\in C} \rho_{ij}\beta_{ij}\frac{W_j+I_{Res_j}}{N_j} + \omega(1-\eta_2)W_i - \nu I_{Res_i} - \frac{1}{\frac{1}{\tau_A}+\delta}\xi_G\xi_R\lambda_A\eta_2 I_{Res_i} \\
&- \frac{1}{\frac{1}{\tau_S}+\delta}(\xi_G\xi_R\eta_2 + \lambda_S(\xi_G(1-\xi_R)+(1-\xi_G))I_{Res_i} - \alpha I_{Res_i} - \gamma I_{Res_i} + \gamma N_i \sum_{j\in C} I_{Res_j}, \\
\dot{W_i} =& \frac{1}{\frac{1}{\tau_S}+\delta}\lambda_S(\xi_G(1-\xi_R)+(1-\xi_G))I_{Res_i} - \nu W_i - \omega W_i - \alpha W_i - \gamma W_i + \gamma N_i \sum_{j\in C} W_j,
\end{aligned}$$

where $i \in C$ denotes that there is a sexual activity classes L with low and a sexual activity class H with high partner change rate. Each sexual activity class N_i includes S_i, susceptible individuals, I_{Sen_i}, individuals infected with antibiotic-sensitive gonorrhoea, I_{Res_i}, individuals infected with gonorrhoea resistant to the first-line antibiotic, and W_i, individuals infected with gonorrhoea resistant to the first-line antibiotic and waiting for re-treatment.

We accounted for heterogeneity in sexual behavior [2] by allowing redistribution of individuals at rate γ. Redistribution is proportional to the size of the sexual activity class, which means that individuals can be redistributed to the same or the other sexual activity class, and individuals from the larger sexual activity class are less likely to change sexual behavior. We accounted for aging by allowing individuals to leave or enter the population at rate α. Susceptible individuals can become infected after contact with an infected individual. Infection thus depends on the transmission probability per partnership, β_{ij}, the partner change rate π_i, and the sexual mixing matrix ρ_{ij}. The sexual mixing matrix ρ_{ij} describes how many partnerships occur within and outside a sexual activity class:

$$\rho_{ij} = \varepsilon\delta_{ij} + (1-\varepsilon)\frac{\pi_j N_j}{\sum_{k\in C}\pi_k N_k},$$

where $\delta_{ij} = 1$ if $i = j$ and zero otherwise. ε is the sexual mixing coefficient [3] which ranges from random or proportionate mixing ($\varepsilon = 0$) to assortative mixing ($\varepsilon = 1$, partnerships only occur within activity classes). All infected individuals can recover spontaneously at rate ν. Individuals infected with asymptomatic, sensitive gonorrhoea are successfully treated at rate $\frac{1}{1/\tau_A+\delta}$ if the test detects gonorrhoea (probability ξ_G), they return for treatment (probability λ_A), and the first-line antibiotic they receive is efficacious (probability η_1). Individuals infected with symptomatic, sensitive gonorrhoea are successfully treated at rate τ_S if the first-line antibiotic they received is efficacious (probability η_1). Individuals infected with asymptomatic, resistant gonorrhoea are successfully treated at rate $\frac{1}{1/\tau_A+\delta}$ if the test detects gonorrhoea (probability ξ_G), the test detects resistance (probability ξ_R), they return for treatment (probability λ_A), and the second-line antibiotic they receive is efficacious (probability η_2). Individuals infected with symptomatic, resistant gonorrhoea are successfully treated at their second visit at rate $\frac{1}{1/\tau_S+\delta}$, if the test detects gonorrhoea (probability ξ_G), the test detects resistance (probability ξ_R), and the second-line antibiotic they receive is efficacious (probability η_2). If either test was unsuccessful, they receive an inefficacious antibiotic at their second visit and if they remain symptomatic (probability λ_S), they enter the waiting compartment W_i. Individuals in W_i are successfully treated with rate ω if the second-line antibiotic they receive is efficacious (probability η_2). If the antibiotic was not efficacious, they remain asymptomatically infected and re-enter the I_{Res} compartment where they might seek care again. We assumed that all individuals whose treatment was not efficacious remain infected and do not again seek care immediately, because treatment is most likely not efficacious for pharyngeal gonorrhoea infections which are usually asymptomatic [4].

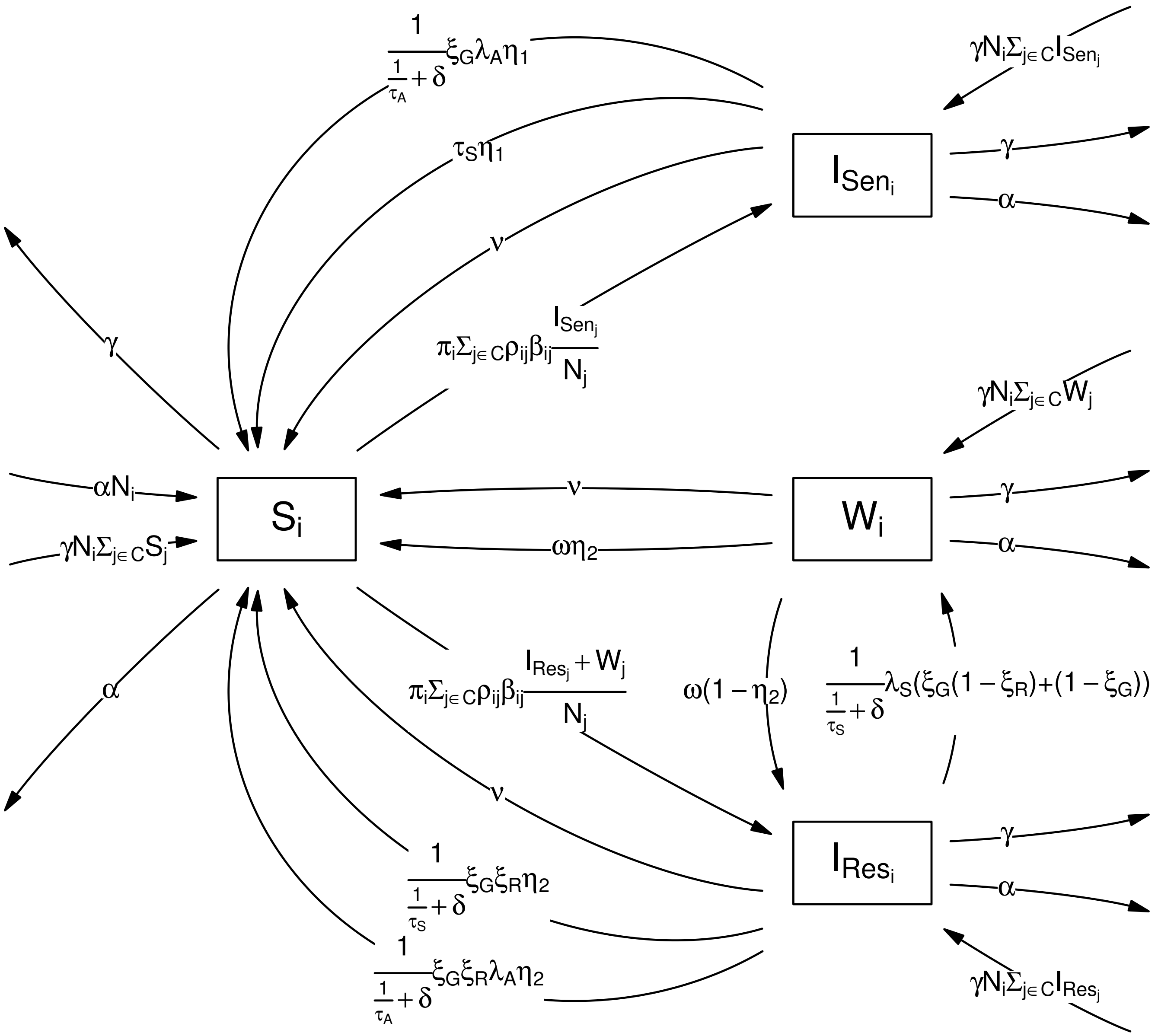

Figure S1. Structure of gonorrhoea transmission, testing and treatment model. N_i: all individuals of sexual activity class i, S_i: individuals of sexual activity class i susceptible to gonorrhoea infection, I_{Sen_i}: individuals of sexual activity class i infected with gonorrhoea sensitive to the first-line antibiotic, I_{Res_i}: individuals of sexual activity class i infected with gonorrhoea resistant to the first-line antibiotic, W_i: individuals of sexual activity class i infected with gonorrhoea resistant to the first-line antibiotic and waiting for re-treatment, π_i: sexual partner change rate, β_{ij}: transmission probability per partnership, ρ_{ij}: mixing between and within sexual activity groups, ν: spontaneous recovery rate, α: rate of entering and leaving the population, γ: redistribution rate, C: set of low and high sexual activity classes, τ_A: rate at which asymptomatic individuals seek care, τ_S: rate at which symptomatic individuals seek care, δ: average time after test individuals return for treatment, $1/\omega$: average time individuals with resistant gonorrhoea wait for re-treatment, λ_A: fraction of asymptomatic individuals who return for treatment, λ_S: fraction of symptomatic individuals who remain symptomatic after failed treatment, ξ_G: test sensitivity to detect gonorrhoea, ξ_R: test sensitivity to detect resistance against the first-line antibiotic, η_1: efficacy of first-line antibiotic, η_2: efficacy of second-line antibiotic.

Table S1. Description of model variables.

Variable	Description
S_i	Individuals of sexual activity class i, susceptible for infection
I_{Sen_i}	Individuals of sexual activity class i, infected with sensitive gonorrhoea
I_{Res_i}	Individuals of sexual activity class i, infected with gonorrhoea resistant to the first-line antibiotic
W_i	Individuals of sexual activity class i, infected with resistant gonorrhoea and waiting for re-treatment
N_i	All individuals of sexual activity class i

Table S2. Description of model parameters. Unless a value is set by definition, all values listed are default values and are varied in sensitivity analyses. Baseline: resistance-free scenario (corresponds to scenario where culture or nucleic acid amplification test (NAAT) is used; ξ_R can take any value since there is no resistance to detect). Culture, NAAT, Point-of-care (POC) with resistance detection (POC+R) and without resistance detection (POC−R) refer to scenarios after resistance is introduced. Sources for parameters: [1] [1], [2] [2], [3] Derived in Derivation of τ_A and τ_S, [4] [5], [5] Assumption, [6] by definition, [7] [6, 7], [8] [8].

Parameter	Description (unit)	Baseline	Culture	NAAT	POC+R	POC−R
β_{ij}	Transmission probability per partnership between activity classes i and j	variable[1]	see Baseline	see Baseline	see Baseline	see Baseline
ρ_{ij}	Sexual mixing between activity class i and j	variable[1]	see Baseline	see Baseline	see Baseline	see Baseline
ν	Spontaneous recovery rate (y^{-1})	variable[1]	see Baseline	see Baseline	see Baseline	see Baseline
π_L	Sexual partner change rate of low activity class (y^{-1})	0.41 (MSM), 0.25 (HMW)[1]	see Baseline	see Baseline	see Baseline	see Baseline
π_H	Sexual partner change rate of high activity class (y^{-1})	30.49 (MSM), 4.57(HMW)[1]	see Baseline	see Baseline	see Baseline	see Baseline
α	Migration rate in and out of the population (y^{-1})	1/29[1]	see Baseline	see Baseline	see Baseline	see Baseline
γ	Rate of redistribution into activity classes (y^{-1})	1[1,2]	see Baseline	see Baseline	see Baseline	see Baseline
τ_S	Rate at which symptomatic individuals seek care (y^{-1})	variable[3]	see Baseline	see Baseline	see Baseline	see Baseline
τ_A	Rate at which asymptomatic individuals seek care (y^{-1})	variable[3]	see Baseline	see Baseline	see Baseline	see Baseline
ξ_G	Test sensitivity to detect gonorrhoea	99%[4]	see Baseline	see Baseline	see Baseline	see Baseline
ξ_R	Test sensitivity to detect resistance against the first-line antibiotic	any value	99%[5]	0%[6]	99%[5]	0%[6]
η_1, η_2	Efficacy of first-line (1) or second-line (2) antibiotic	99%[7]	see Baseline	see Baseline	see Baseline	see Baseline
δ	Average time after test individuals return for treatment (days)	7[8]	see Baseline	see Baseline	0[6]	0[6]
$1/\omega$	Average time individuals with resistant gonorrhoea wait for re-treatment (days)	7[5]	see Baseline	see Baseline	see Baseline	see Baseline
λ_A	Fraction of asymptomatic individuals who return for treatment	90%[5]	see Baseline	see Baseline	100%[6]	100%[6]
λ_S	Fraction of symptomatic individuals who remain symptomatic after failed treatment	90%[5]	see Baseline	see Baseline	see Baseline	see Baseline
ψ	Fraction of successfully treated individuals who were symptomatic at baseline	60%[8]	–	–	–	–

2. Derivation of τ_A and τ_S

Our previous gonorrhoea transmission model included a single treatment rate, τ, describing the rate of recovery for all individuals that received treatment [1]. Here, we decomposed τ into the rate of successful treatment for asymptomatic individuals (i.e. the rate of successful treatment following screening or partner notification (PN)), τ'_A, and the rate of successful treatment for symptomatic individuals, τ'_S:

$$\tau = \tau'_A + \tau'_S$$

The extended model distinguishes between the rates at which asymptomatic (τ_A) or symptomatic (τ_S) individuals seek care, and the subsequent processes that determine whether and when treatment was given (ξ_G, λ_A, δ) and whether it was successful (η_1). Note that we derived τ_A and τ_S for the baseline scenario without resistance and thus did not take resistance or the second-line antibiotic into account. The overall rate of successful treatment for asymptomatic individuals in our model is thus

$$\tau'_A = \frac{1}{\frac{1}{\tau_A} + \delta_{\text{baseline}}} \xi_G \lambda_{A,\text{ baseline}} \eta_1 \quad ,$$

and the rate of successful treatment for symptomatic patients is

$$\tau'_S = \tau_S \eta_1$$

We introduced the parameter ψ, the fraction of successfully treated individuals who were symptomatic at baseline and can derive τ_A and τ_S:

$$\psi = \frac{\tau'_S}{\tau} = \frac{\tau_S \eta_1}{\tau}, \quad \tau_S = \frac{\psi \tau}{\eta_1}$$

and

$$\tau'_A = \tau - \tau'_S = \tau(1-\psi) = \frac{1}{\frac{1}{\tau_A} + \delta_{\text{baseline}}} \xi_G \lambda_{A,\text{ baseline}} \eta_1,$$

$$\tau_A = \frac{\tau(1-\psi)}{\xi_G \lambda_{A,\text{ baseline}} \eta_1 - \delta_{\text{baseline}} \tau(1-\psi)} \quad .$$

If the combination of calibrated parameter set and diagnosis and treatment parameters lead to negative values of τ_A, the calibrated parameter set was excluded from analysis for these diagnosis and treatment parameters.

3. Prevalence and incidence rates before resistance

In our previous study, we calibrated the sexual mixing coefficient ε, the fraction of diagnosed and treated infections ϕ, the average duration of infection $D = \phi/\tau$, the transmission probability within the low activity class β_{LL}, and the transmission probability within the high activity class β_{HH} to yield prevalence and incidence of diagnosed and treated infections (calculated for activity class i with $\phi S_i \pi_i \sum_{j \in C} \rho_{ij} \beta_{ij} (I_{Sen_j} + I_{Res_j} + W_j)/N_j$ per year) within empirically observed ranges (Table 3 and 4 in the main text) [1]. In this study, we used subsets of 1 000 calibrated parameter sets from the previous study to simulate men who have sex with men (MSM) and heterosexual men and women (HMW) populations. The distributions of prevalence and incidence of diagnosed and treated infection in the resistance-free equilibrium (i.e. before resistance is introduced) based on 1 000 calibrated parameter sets are shown in Fig. S2 and S3.

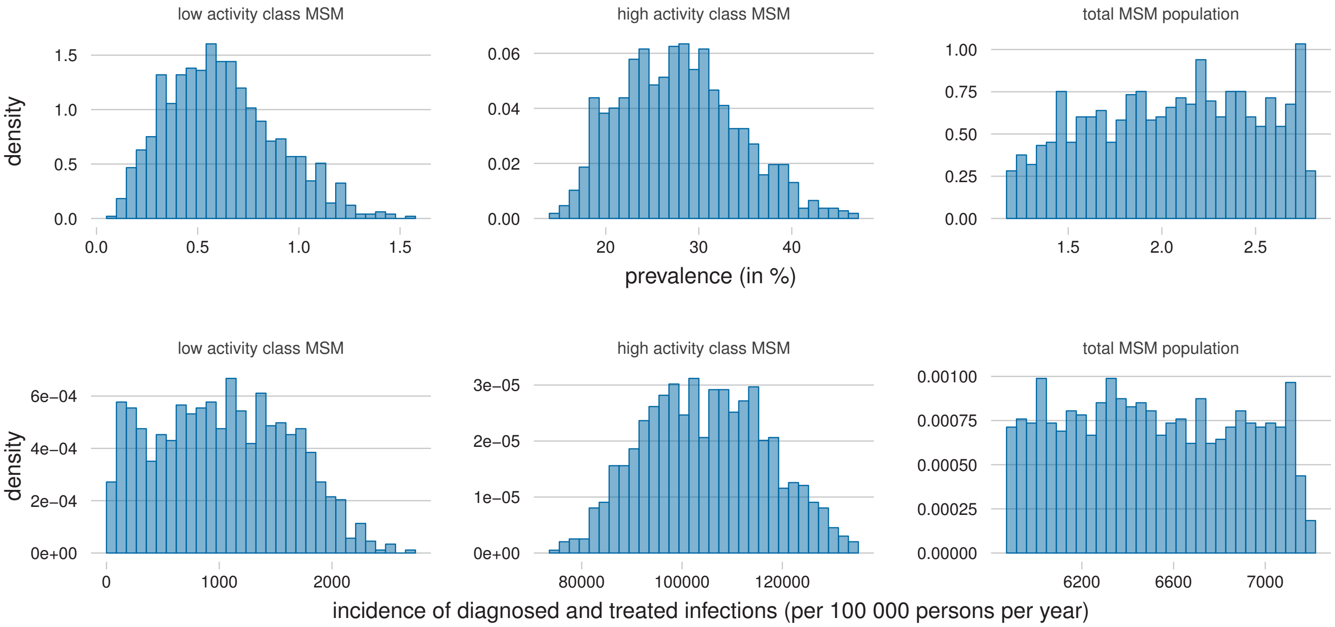

Figure S2. Prevalence and incidence of diagnosed and treated infections in men who have sex with men (MSM) before resistance is introduced. In our previous study, we calibrated five parameters to yield model simulations with prevalences and incidences of diagnosed and treated infections within a range empirically observed in the Health in Men (HIM) Study in Australia [9] (Table 3 in the main text). In this study, we used a subset of 1 000 calibrated parameter sets to simulate a population of MSM. Shown are the distributions of prevalences and incidences of diagnosed and treated infections for low activity class MSM, high activity class MSM, and the total MSM population.

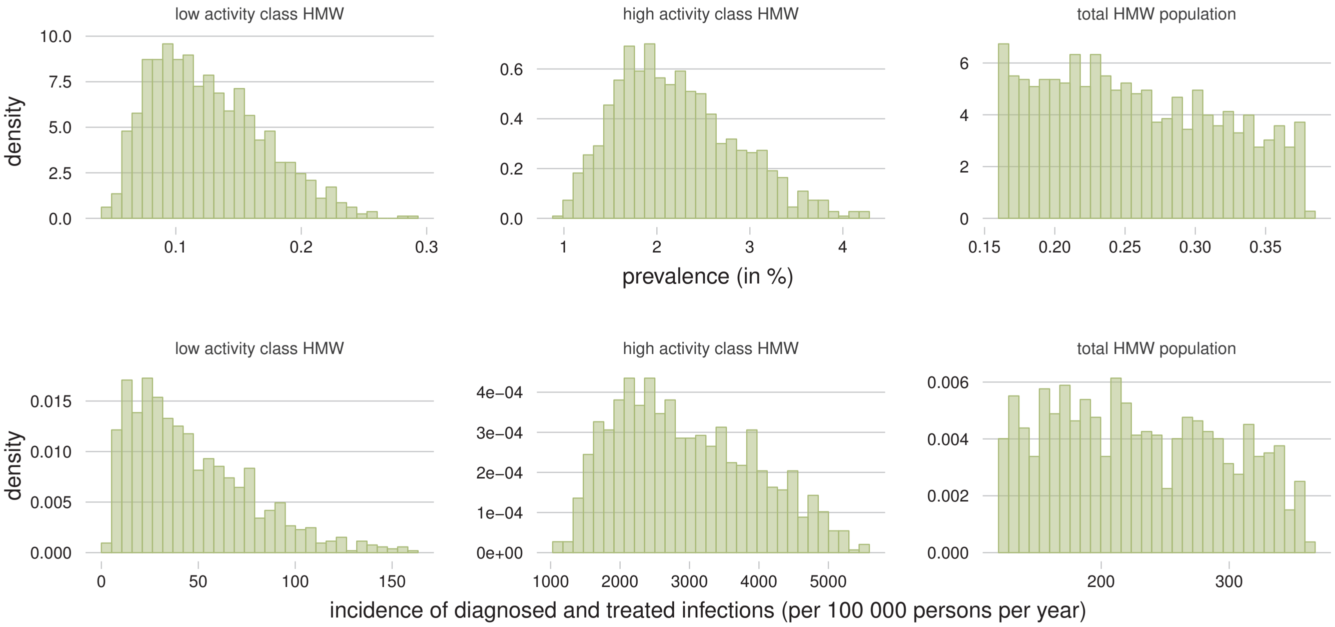

Figure S3. Prevalence and incidence of diagnosed and treated infections in heterosexual men and women (HMW) before resistance is introduced. In our previous study, we calibrated five parameters to yield model simulations with prevalences and incidences of diagnosed and treated infections within a range empirically observed in the National Health and Nutrition Examination Survey (NHANES) [10] and surveillance data [11], both from the Centers for Disease Control (CDC) (Table 4 in the main text). In this study, we used a subset of 1 000 calibrated parameter sets to simulate a population of HMW. Shown are the distributions of prevalences and incidences of diagnosed and treated infections for low activity class HMW, high activity class HMW, and the total HMW population.

4. Rate of resistance spread and ratio of resistance spread

The rate at which resistance spreads can be measured as the slope of the ratio of resistant and sensitive infections over time. We estimated the slope by fitting linear growth models (function *lm* in R language and software environment for statistical computing [12]) to the log transformed ratio of resistant and sensitive infections over time. Some parameter sets lead to complete eradication of gonorrhoea from the population (Fig. S4). Since resistance cannot spread when gonorrhea is extinct, we did not calculate the ratio of the rate of resistance spread for these parameter sets.

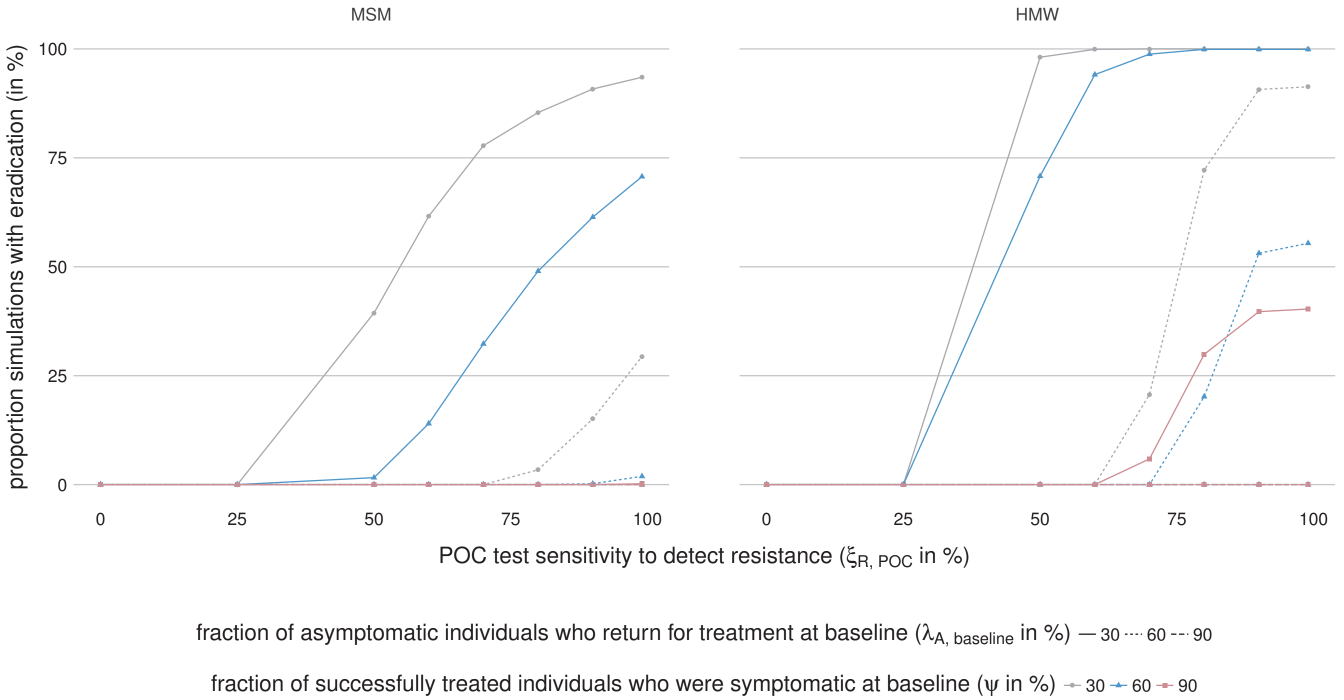

Figure S4. Eradication of gonorrhoea is more likely when sensitivity to detect resistance is high. Each parameter combination of $\lambda_{A,\text{ baseline}}$, ψ and $\xi_{R,\text{ POC}}$ was simulated with 1 000 calibrated parameter sets. The plots show for each parameter combination of $\lambda_{A,\text{ baseline}}$, ψ and $\xi_{R,\text{ POC}}$ in how many simulations with different calibrated parameter sets gonorrhoea was eradicated. POC: point-of-care, MSM: men who have sex with men, HMW: heterosexual men and women.

5. Sensitivity analyses

We performed one-dimensional sensitivity analyses of the observed cases averted regarding ξ_G, ξ_R, $\lambda_{A,\text{ baseline}}$, λ_S, ψ, δ_{baseline}, ω (Fig. S5-S11). As described in the main text, observed cases averted are defined as the difference between the cumulative incidence of observed (i.e. diagnosed and successfully treated at baseline; fraction ϕ [1]) cases using NAAT and the cumulative incidence of observed cases using culture or POC tests. We calculated the observed cases averted 5 years after the introduction of resistance.

We also simulated an alternative baseline scenario where culture has a lower test sensitivity to detect gonorrhoea and only culture is used at baseline ($\xi_{G,\text{ baseline}} = \xi_{G,\text{ culture}} = 90\%$, all other values as in Table S2, Fig. S12-S14).

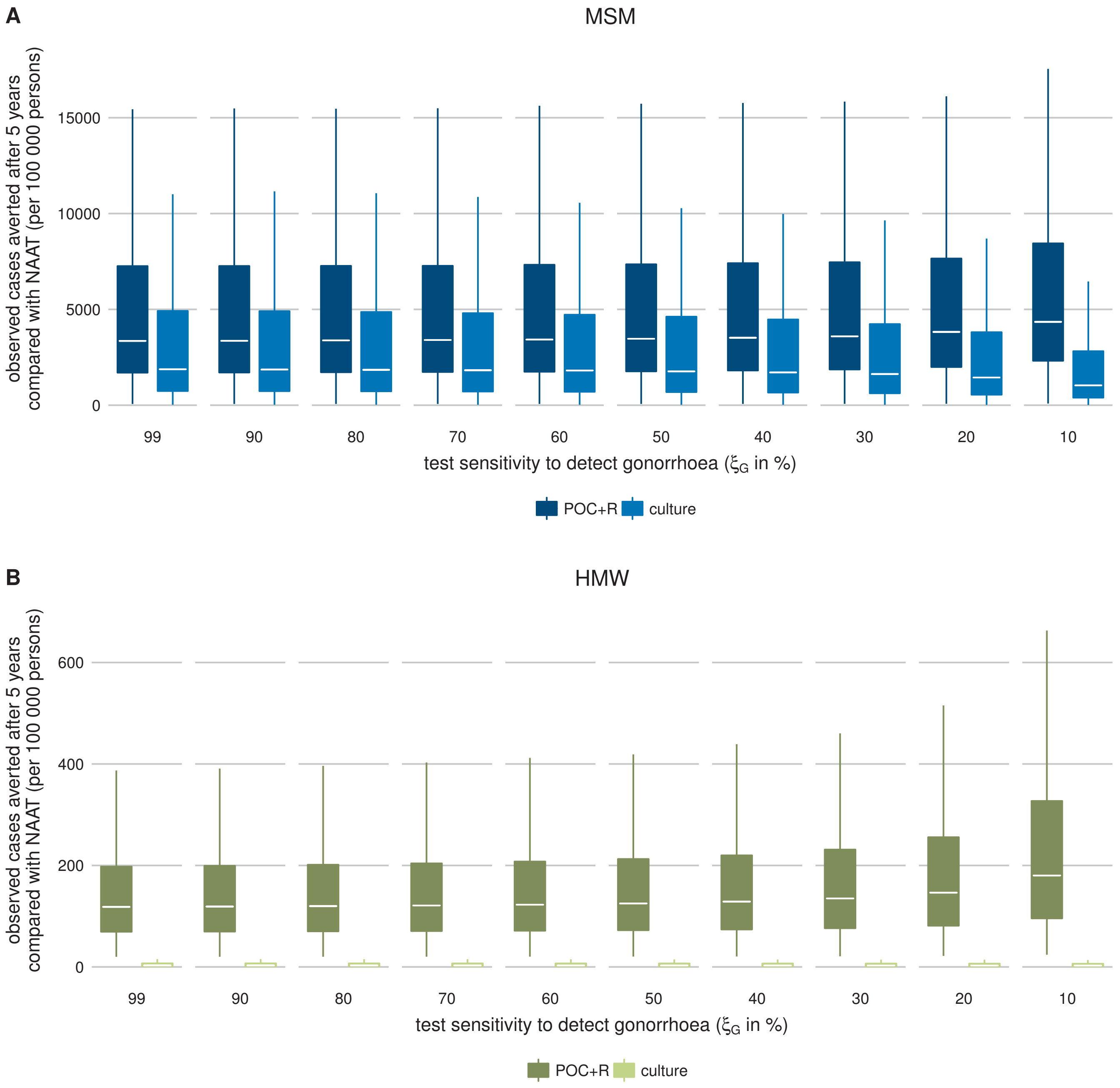

Figure S5. One-dimensional sensitivity analysis of observed cases averted (per 100000 persons) using POC or culture compared with NAAT with respect to the test sensitivity to detect gonorrhoea, ξ_G, for (A) men who have sex with men (MSM) and (B) heterosexual men and women (HMW). The default value for $\xi_G = \xi_{G,\text{ baseline}} = \xi_{G,\text{ culture}} = \xi_{G,\text{ NAAT}} = \xi_{G,\text{ POC}}$ is 99%. NAAT: nucleic acid amplification test, POC+R: point-of-care test with resistance detection. Lower/upper bound of the box indicate first/third quartiles, bar in box indicates median, whiskers span 1.5 times interquartile range. Outliers not shown for more clarity.

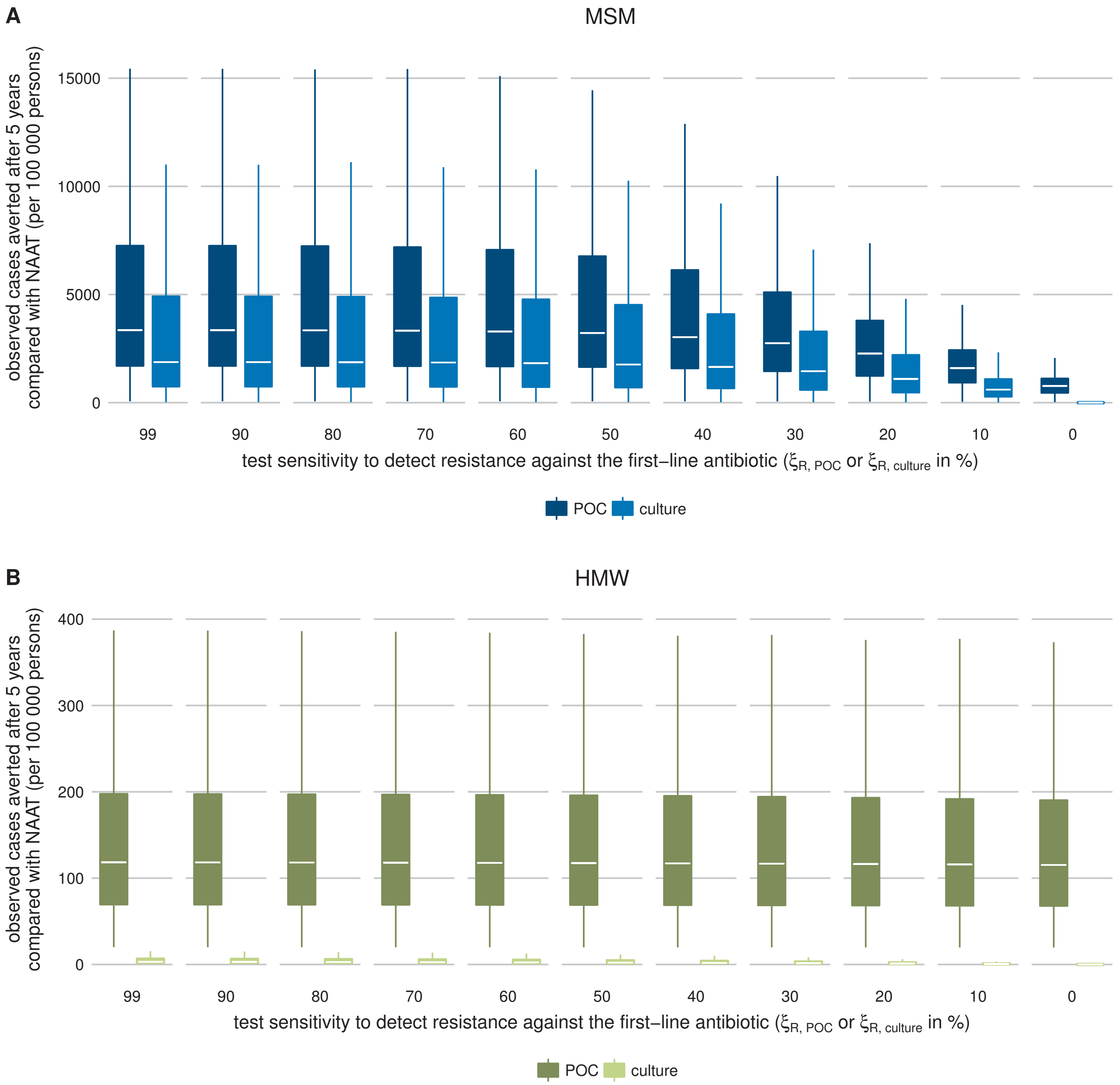

Figure S6. One-dimensional sensitivity analysis of observed cases averted (per 100 000 persons) using POC or culture compared with NAAT with respect to the test sensitivity to detect resistance against the first-line antibiotic, ξ_R, for (A) men who have sex with men (MSM) and (B) heterosexual men and women (HMW). The default value for $\xi_{R,\text{ baseline}} = \xi_{R,\text{ culture}} = \xi_{R,\text{ POC}}$ is 99% (and $\xi_{R,\text{ NAAT}} = 0\%$ by definition). NAAT: nucleic acid amplification test, POC+R: point-of-care test with resistance detection. Lower/upper bound of the box indicate first/third quartiles, bar in box indicates median, whiskers span 1.5 times interquartile range. Outliers not shown for more clarity.

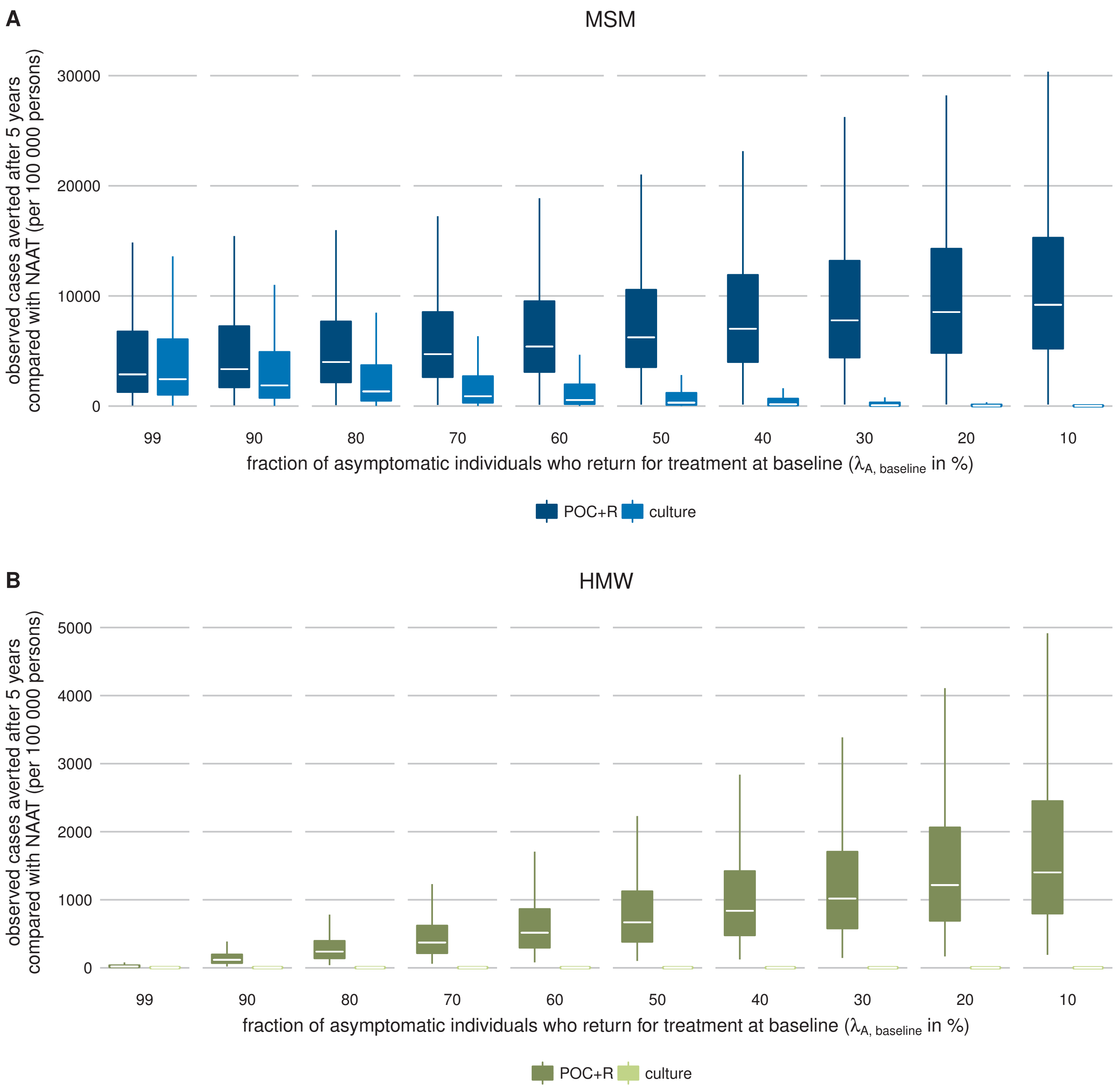

Figure S7. One-dimensional sensitivity analysis of observed cases averted (per 100 000 persons) using POC or culture compared with NAAT with respect to the fraction of asymptomatic individuals who return for treatment at baseline, $\lambda_{A,\text{ baseline}}$, for (A) men who have sex with men (MSM) and (B) heterosexual men and women (HMW). The default value for $\lambda_{A,\text{ baseline}} = \lambda_{A,\text{ culture}} = \lambda_{A,\text{ NAAT}}$ is 90% (and $\lambda_{A,\text{ POC}} = 100\%$ by definition). NAAT: nucleic acid amplification test, POC+R: point-of-care test with resistance detection. Lower/upper bound of the box indicate first/third quartiles, bar in box indicates median, whiskers span 1.5 times interquartile range. Outliers not shown for more clarity.

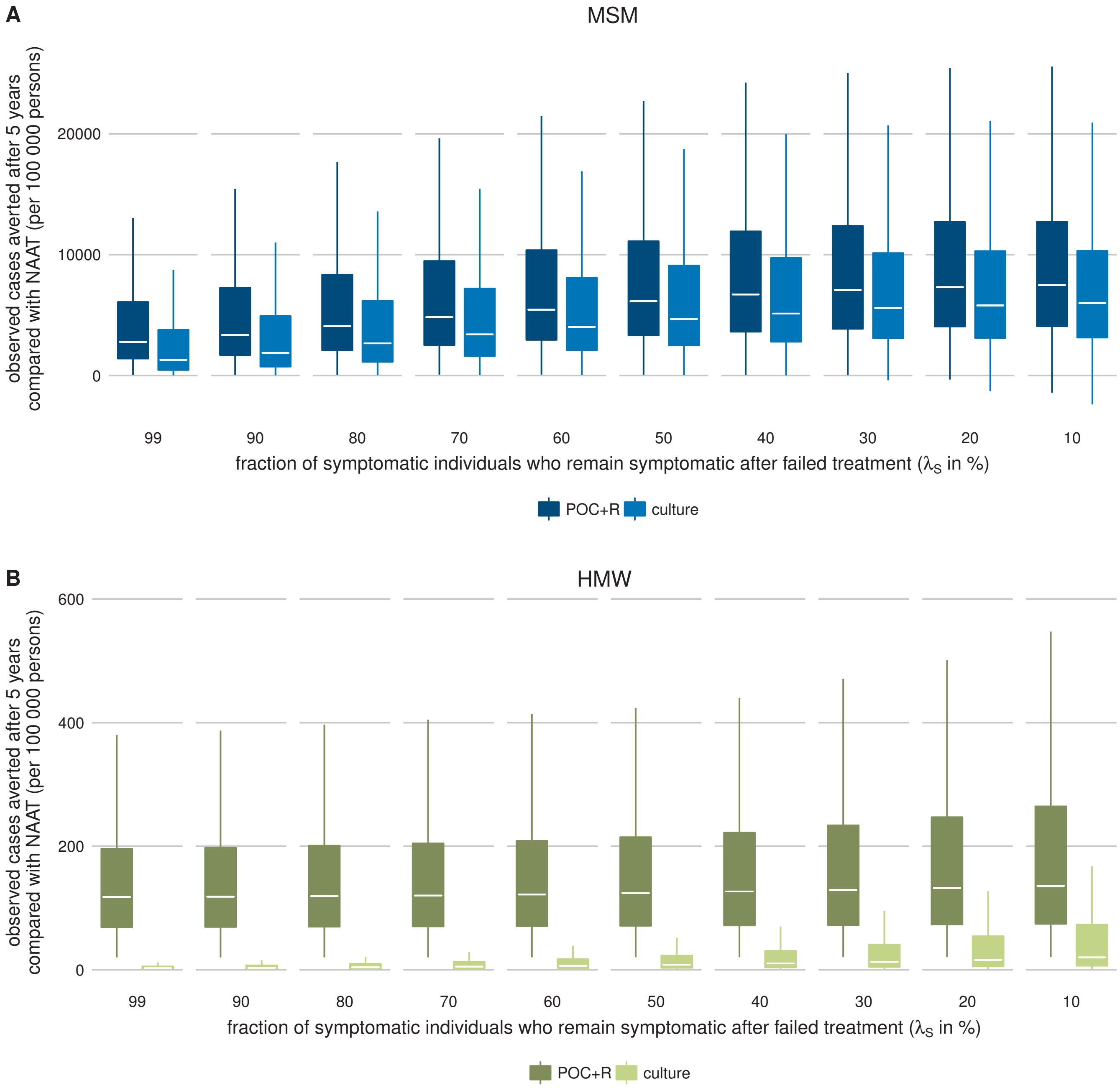

Figure S8. One-dimensional sensitivity analysis of observed cases averted (per 100 000 persons) using POC or culture compared with NAAT with respect to the fraction of symptomatic individuals who remain symptomatic after failed treatment, λ_S, for (A) men who have sex with men (MSM) and (B) heterosexual men and women (HMW). The default value for $\lambda_S = \lambda_{S,\text{ baseline}} = \lambda_{S,\text{ culture}} = \lambda_{S,\text{ NAAT}} = \lambda_{S,\text{ POC}}$ is 90%. NAAT: nucleic acid amplification test, POC+R: point-of-care test with resistance detection. Lower/upper bound of the box indicate first/third quartiles, bar in box indicates median, whiskers span 1.5 times interquartile range. Outliers not shown for more clarity.

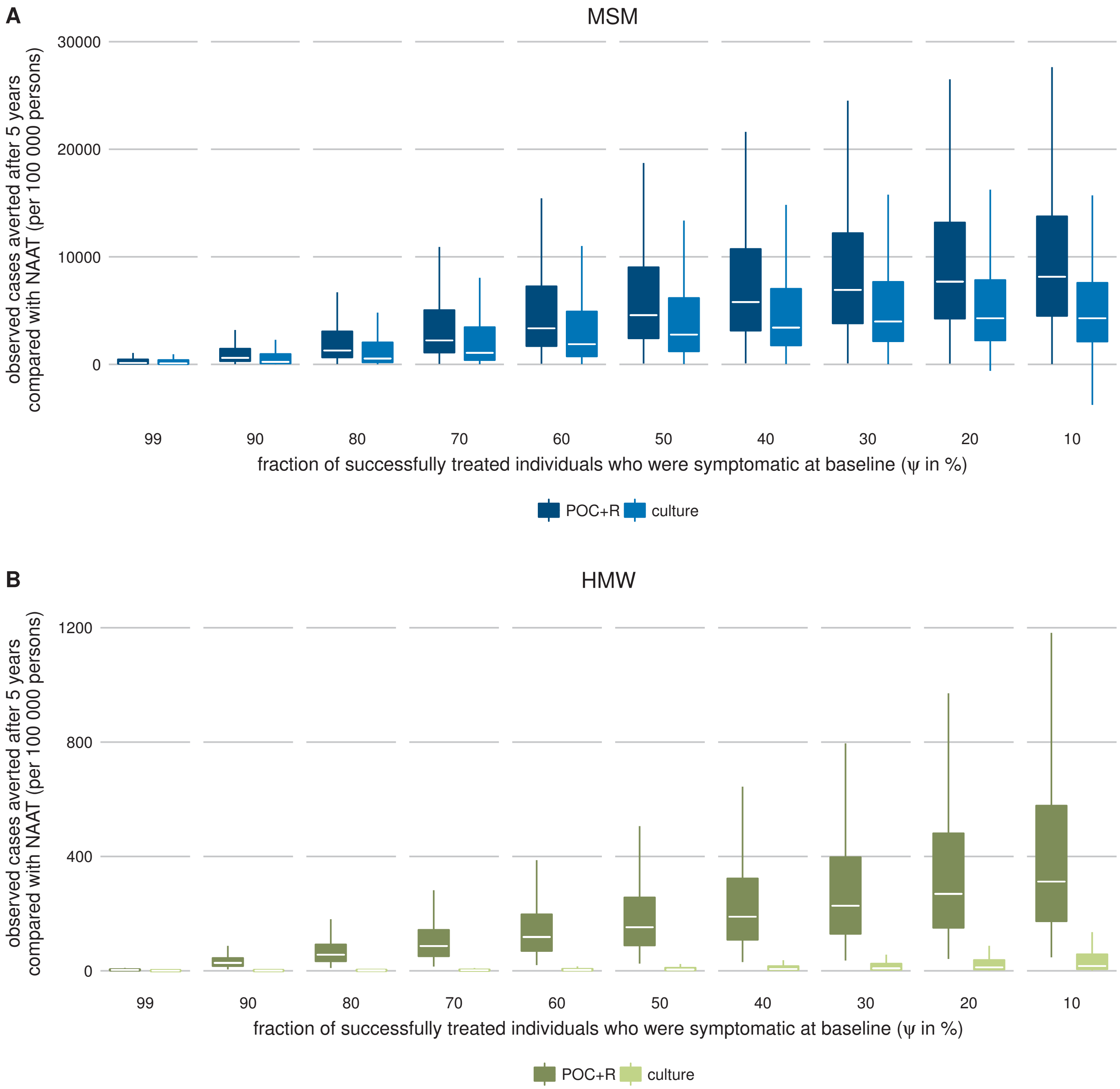

Figure S9. One-dimensional sensitivity analysis of observed cases averted (per 100 000 persons) using POC or culture compared with NAAT with respect to the fraction of successfully treated individuals who were symptomatic at baseline, ψ, for (A) men who have sex with men (MSM) and (B) heterosexual men and women (HMW). The default value for ψ is 60%. NAAT: nucleic acid amplification test, POC+R: point-of-care test with resistance detection. Lower/upper bound of the box indicate first/third quartiles, bar in box indicates median, whiskers span 1.5 times interquartile range. Outliers not shown for more clarity.

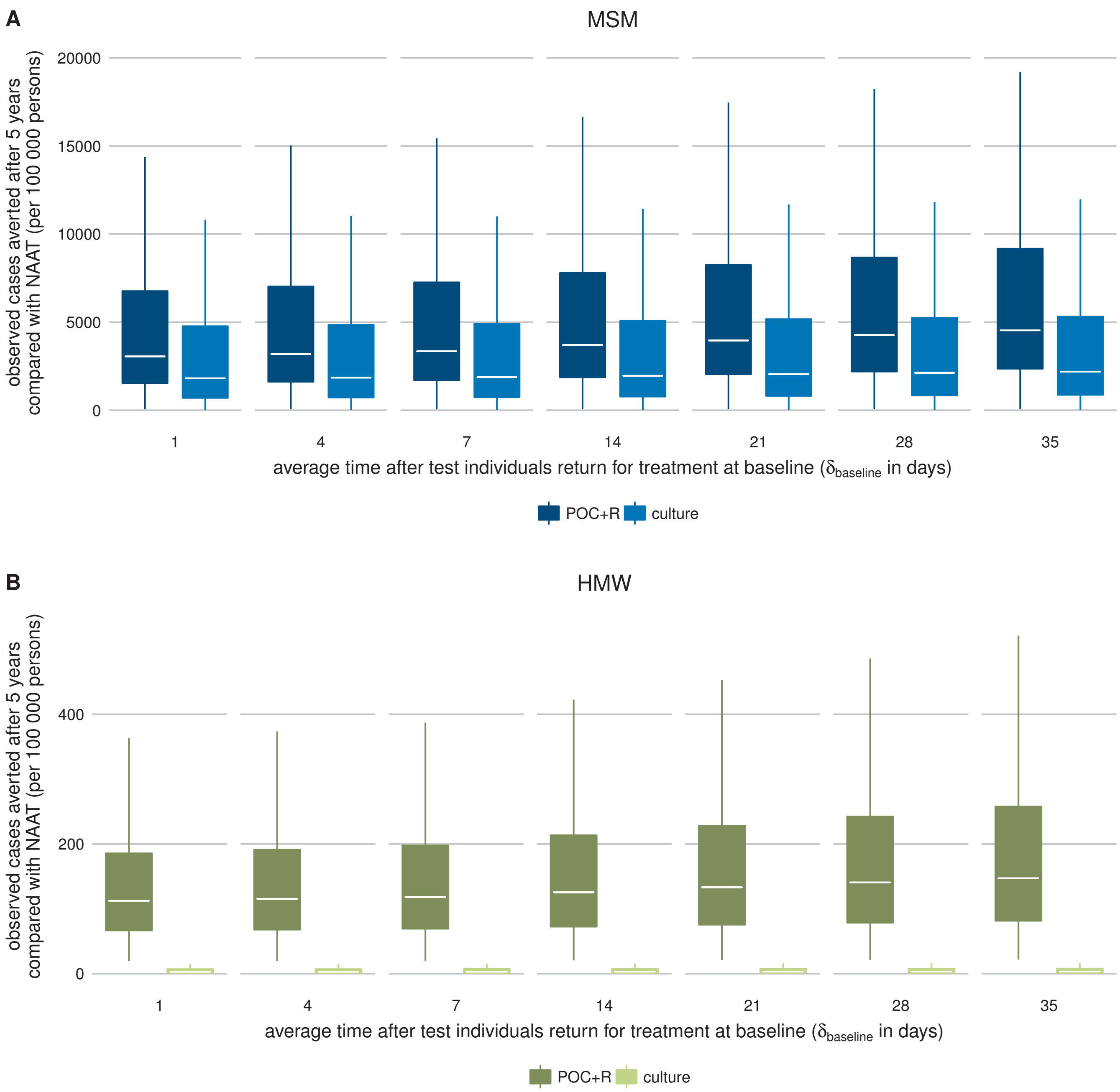

Figure S10. One-dimensional sensitivity analysis of observed cases averted (per 100 000 persons) using POC or culture compared with NAAT with respect to the average time after test individuals return for treatment at baseline, δ_{baseline}, for (A) men who have sex with men (MSM) and (B) heterosexual men and women (HMW). The default value for $\delta_{\text{baseline}} = \delta_{\text{culture}} = \delta_{\text{NAAT}}$ is 7 days (and $\delta_{\text{POC}} = 0$ days by definition). NAAT: nucleic acid amplification test, POC + R: point-of-care test with resistance detection. Lower/upper bound of the box indicate first/third quartiles, bar in box indicates median, whiskers span 1.5 times interquartile range. Outliers not shown for more clarity.

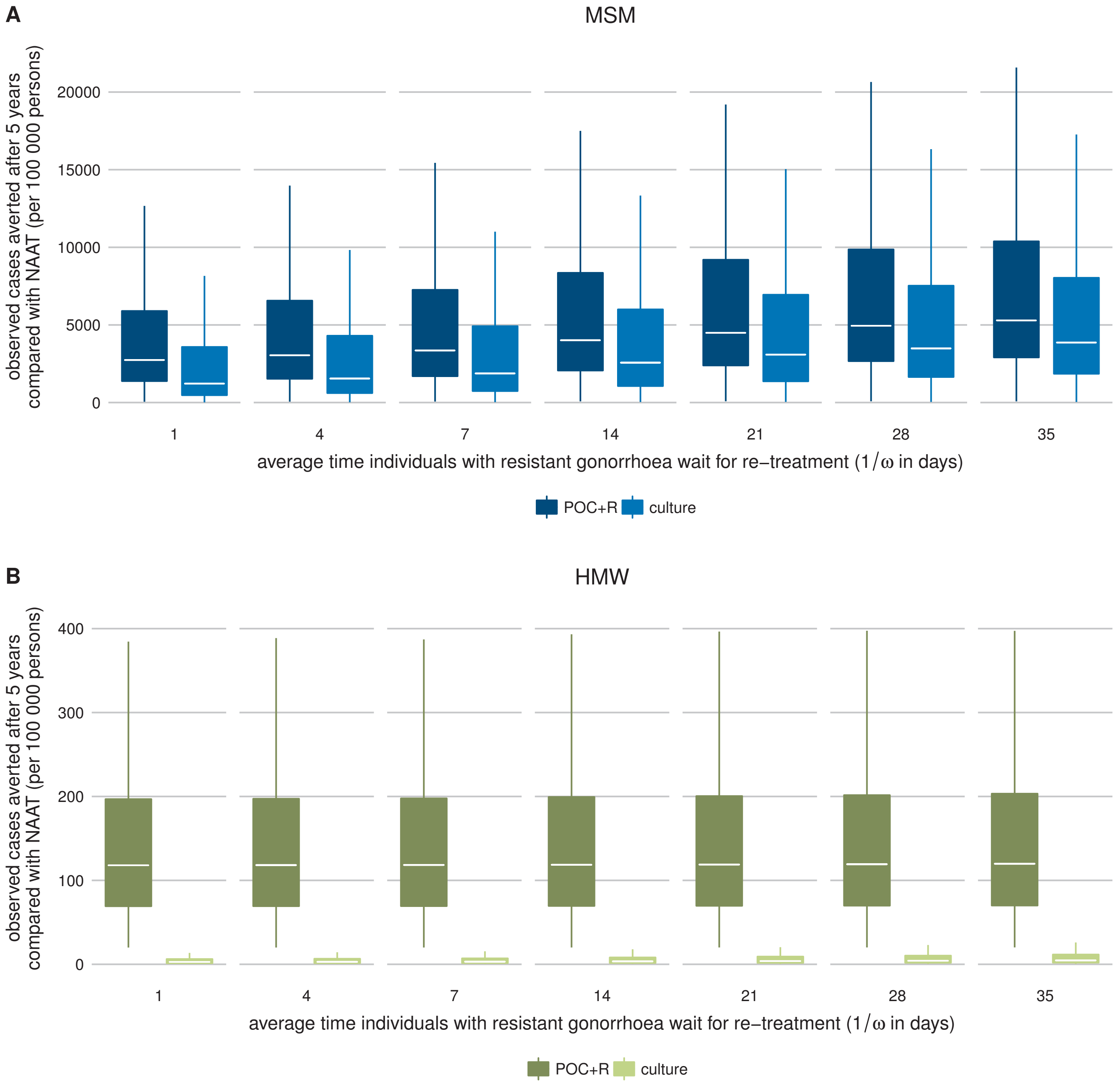

Figure S11. One-dimensional sensitivity analysis of observed cases averted (per 100 000 persons) using POC or culture compared with NAAT with respect to the average time individuals with resistant gonorrhoea wait for re-treatment, $1/\omega$, for (A) men who have sex with men (MSM) and (B) heterosexual men and women (HMW). The default value for $1/\omega = 1/\omega_{\text{baseline}} = 1/\omega_{\text{culture}} = 1/\omega_{\text{NAAT}} = 1/\omega_{\text{POC}}$ is 7 days. NAAT: nucleic acid amplification test, POC+R: point-of-care test with resistance detection. Lower/upper bound of the box indicate first/third quartiles, bar in box indicates median, whiskers span 1.5 times interquartile range. Outliers not shown for more clarity.

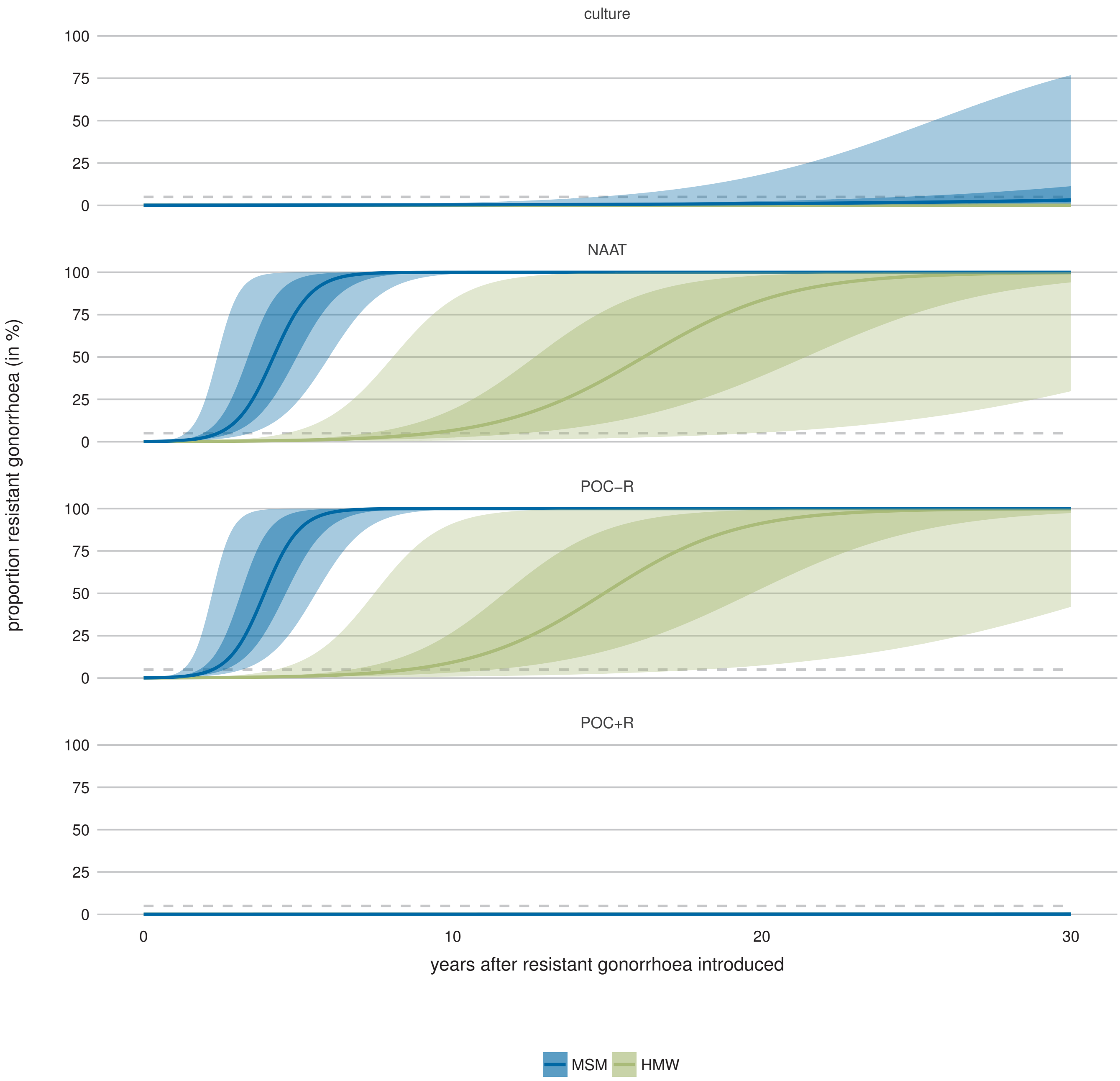

Figure S12. Timeline of the proportion of resistant gonorrhoea infections when using culture, NAAT, POC − R or POC + R, assuming that culture has a lower test sensitivity to detect gonorrhoea and only culture is used at baseline ($\xi_{G,\text{ baseline}} = \xi_{G,\text{ culture}} = 90\%$, all other values as in Table S2). Lower ξ_G requires a higher rate at which asymptomatic individuals seek care (τ_A) to obtain the same prevalence and incidence rates at baseline (see Derivation of τ_A and τ_S). NAAT: nucleic acid amplification test, POC − R: point-of-care test (POC) without resistance detection, POC + R: POC with resistance detection, MSM: men who have sex with men, HMW: heterosexual men and women.

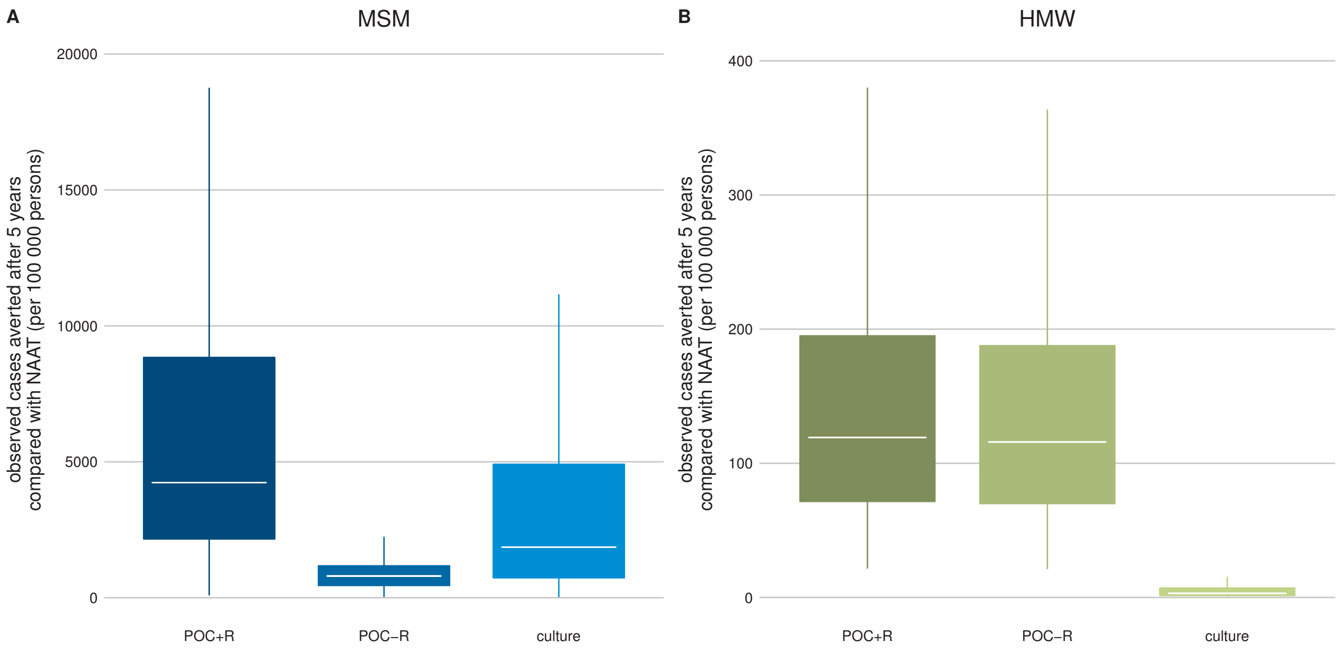

Figure S13. Observed cases averted after 5 years compared with nucleic acid amplification test (NAAT) (per 100 000 persons) in (A) men who have sex with men (MSM) and (B) heterosexual men and women (HMW), assuming that culture has a lower test sensitivity to detect gonorrhoea and only culture is used at baseline ($\xi_{G,\text{ baseline}} = \xi_{G,\text{ culture}} = 90\%$, all other values as in Table S2). POC + R: point-of-care test (POC) with resistance detection, POC − R: POC without resistance detection. Lower ξ_G requires a higher rate at which asymptomatic individuals seek care (τ_A) to obtain the same prevalence and incidence rates at baseline (see Derivation of τ_A and τ_S).

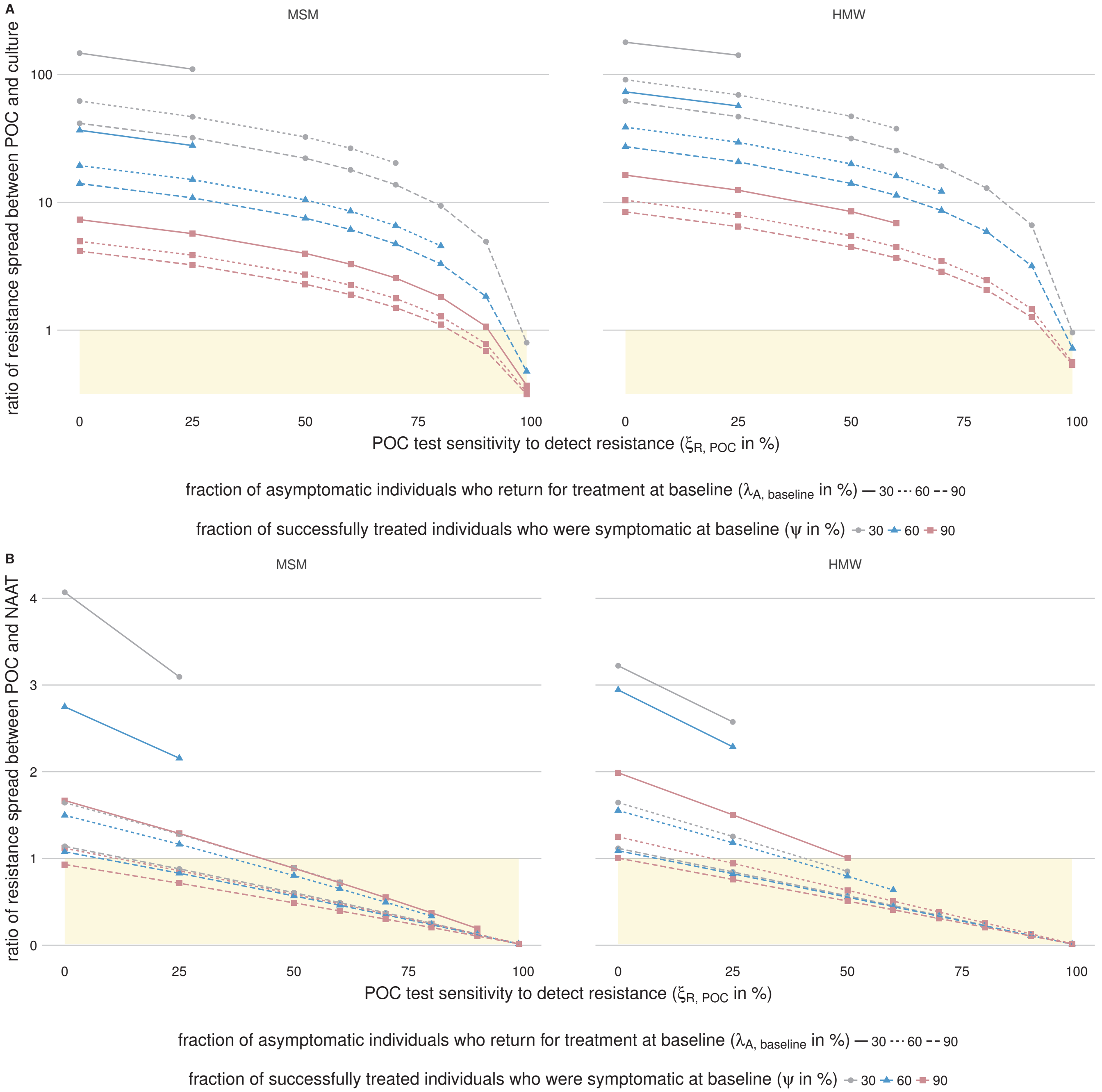

Figure S14. Three-dimensional sensitivity analysis of the ratio of resistance spread between between point-of care test (POC) (POC − R if $\xi_{R,\text{ POC}} = 0$ and POC + R if $\xi_{R,\text{ POC}} > 0$) and (A) culture ($\xi_{R,\text{ culture}}$ fixed to 99%) or (B) nucleic acid amplification test (NAAT, $\xi_{R,\text{ NAAT}} = 0$ by definition), assuming that culture has a lower test sensitivity to detect gonorrhoea and only culture is used at baseline ($\xi_{G,\text{ baseline}} = \xi_{G,\text{ culture}} = 90\%$, all other values as in Table S2). Lower ξ_G requires a higher rate at which asymptomatic individuals seek care (τ_A) to obtain the same prevalence and incidence rates at baseline (see Derivation of τ_A and τ_S). The shaded areas indicate that resistance spread is slower when using POC than when using culture. Each data point gives the median value over 1 000 simulations (one per calibrated parameter set). Some calibrated parameter sets lead to extinction of gonorrhoea in the simulation (see Fig. S4). In these simulations, resistance did not spread and the ratio of resistance spread could not be calculated. Data points that would include such simulations were excluded from this figure since they would show the median ratio of resistance spread over less than 1 000 simulations.

References

[1] Fingerhuth, S.M., Bonhoeffer, S., Low, N., Althaus, C.L.: Antibiotic-resistant Neisseria gonorrhoeae spread faster with more treatment, not more sexual partners. PLoS Pathog **12**(5), 1005611 (2016). doi:10.1371/journal.ppat.1005611

[2] Althaus, C.L., Choisy, M., Alizon, S., CSF group: Number of sex acts matters for heterosexual transmission and control of Chlamydia trachomatis. PeerJ PrePrints **3**, 1164 (2015)

[3] Hethcote, H.W., Yorke, J.A., Nold, A.: Gonorrhea modeling: a comparison of control methods. Math Biosci **58**, 93–109 (1982). doi:10.1016/0025-5564(82)90053-0

[4] Dudareva-Vizule, S., Haar, K., Sailer, A., Wisplinghoff, H., Wisplinghoff, F., Marcus, U., PARIS study group: Prevalence of pharyngeal and rectal Chlamydia trachomatis and Neisseria gonorrhoeae infections among men who have sex with men in Germany. Sex Transm Infect **90**(1), 46–51 (2014). doi:10.1136/sextrans-2012-050929

[5] Whiley, D.M., Tapsall, J.W., Sloots, T.P.: Nucleic acid amplification testing for Neisseria gonorrhoeae: an ongoing challenge. J Mol Diagn **8**(1), 3–15 (2006). doi:10.2353/jmoldx.2006.050045

[6] Tapsall, J., World Health Organization: Antimicrobial resistance in Neisseria gonorrhoeae (2001). http://www.who.int/entity/csr/resources/publications/drugresist/Neisseria_gonorrhoeae.pdf

[7] Handsfield, H.H., Dalu, Z.A., Martin, D.H., Douglas, J.M., McCarty, J.M., Schlossberg, D., Azithromycin Gonorrhea Study Group: Multicenter trial of single-dose azithromycin vs. ceftriaxone in the treatment of uncomplicated gonorrhea. Sex Transm Dis **21**(2), 107–11 (1994)

[8] Brook, G.: The performance of non-NAAT point-of-care (POC) tests and rapid NAAT tests for chlamydia and gonorrhoea infections. An assessment of currently available assays. Sex Transm Infect **91**, 539–44 (2015). doi:10.1136/sextrans-2014-051997

[9] Jin, F., Prestage, G.P., Mao, L., Kippax, S.C., Pell, C.M., Donovan, B., Cunningham, P.H., Templeton, D.J., Kaldor, J.M., Grulich, A.E.: Incidence and risk factors for urethral and anal gonorrhoea and chlamydia in a cohort of HIV-negative homosexual men: the Health in Men Study. Sex Transm Infect **83**(2), 113–9 (2007). doi:10.1136/sti.2006.021915

[10] Datta, S.D., Sternberg, M., Johnson, R.E., Berman, S., Papp, J.R., McQuillan, G., Weinstock, H.: Gonorrhea and chlamydia in the United States among persons 14 to 39 years of age, 1999 to 2002. Ann Intern Med **147**(2), 89–96 (2007). doi:10.1016/S0084-3954(08)79138-3

[11] Centers for Disease Control and Prevention: Sexually transmitted disease surveillance 2013. U.S. Department of Health and Human Services, Atlanta (2014)

[12] R Core Team: R: a language and environment for statistical computing. R Foundation for Statistical Computing, Vienna, Austria (2013). http://www.r-project.org/
